# Accelerating Prediction of Complex Molecular Crystals by Sensible Selection of Asymmetric Units

**DOI:** 10.1021/acs.jctc.6c00623

**Published:** 2026-06-23

**Authors:** Jordan A. Dorrell, Graeme M. Day

**Affiliations:** School of Chemistry and Chemical Engineering, 7423 University of Southampton, Southampton, SO17 1BJ, United Kingdom

## Abstract

Effective crystal structure prediction (CSP) relies on thorough exploration of potential energy surfaces. For this reason, molecular CSP has historically focused on simple crystals with a single molecule in the asymmetric unit. Increasing the number of molecules in the asymmetric unit increases the degrees of freedom of the crystal structure and the burden on the crystal structure predictor. We mitigate this burden by modifying quasi-random structure searching (QRSS) with “sensible asymmetric units for crystal exploration” (SAUCE). Compared to traditional QRSS implementations, the crystal structures generated by SAUCE are denser, and in a more energetically favorable environment, leading to faster geometry optimizations and more frequent recovery of the low energy minima discovered by traditional QRSS. This will allow for structure predictors to approach more complex molecular materials and to reduce the computational cost of molecular CSP.

## Graphical abstract

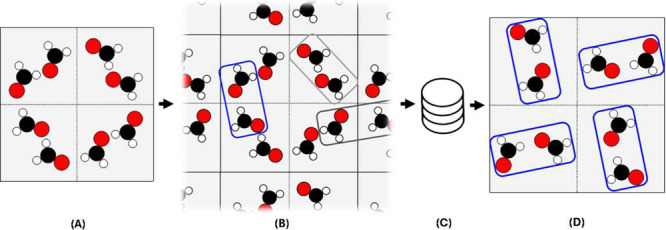

## 1 Introduction

Crystal structure prediction (CSP) is an N-body problem for which there are a vanishingly small number of solutions which are experimentally realizable, making it a notoriously challenging and computationally expensive task. For crystal structure predictors, it is convenient and computationally efficient to describe a crystal structure’s unit cell in terms of its cell parameters (three lengths, and three angles), its space group (and associated symmetry operators), and the coordinates of the atoms within the asymmetric unit (the minimum representation of symmetry-independent atoms). Although it is geometrically possible for a single molecule to be shared between multiple asymmetric units, this is not conducive to symmetry-constrained geometry optimizations and therefore, in practice, the asymmetric unit is usually taken to be the minimum representation of symmetry-independent whole molecules. Within this descriptive framework: the total number of formula units within the unit cell is denoted *Z*, the total number of formula units within the asymmetric unit is denoted *Z*′, and the total number of independent units (molecules or ions) in the asymmetric unit is denoted *G*.^1,2^ For small rigid molecules, CSP is now routine^3^ if the crystal structure contains only one molecule per asymmetric unit. For elements where intermolecular force fields have been well parametrized, CSP for such systems can be fast and reliable.^3,4^ However, molecular CSP still faces challenges in instances where *G* is greater than one.

Almost 40 years after Maddox described the lack of predictibility of crystal structures as a “continuing scandal”,^5^ the field of CSP still faces the same two large barriers to success; there is no known method for directly predicting a structure (i.e., one cannot solve for a crystal structure), and there is no known means to prove whether a crystal structure is the most stable (or stable at all) without comparing it to another crystal. For these reasons, across both molecular and inorganic CSP, the standard approach is to exhaustively search the energy minima of potential energy surfaces (PES). The size of a PES correlates with the numbers of degrees of freedom, *df*, in the system, and may be expressed as in eq 1,

$$df=df\left(l\right)+\overset{G}{\underset{i}{\sum }}\left(3+df{\left(r\right)}_{i}+df{\left(\mathrm{int}ra\right)}_{i}\right)$$ 1

*df* is a sum of several contributing degrees of freedom. For each free unit in the asymmetric units, of which there are *G*, there are three translational degrees of freedom, between one and three rotational degrees of freedom, *df*(*r*), and zero or more additional degrees of freedom from intramolecular flexibility, *df*(intra). Lastly, there is an additional one to six lattice degrees of freedom, *df*(*l*), which is constrained by the space group. For example, *G* = 1, 2, and 3 triclinic crystal structures of rigid, nonlinear molecules have 12, 18, and 24 degrees of freedom, respectively. It is therefore unsurprising that early attempts at molecular CSP were largely constrained to *G* = 1 systems.

The challenge posed by high-*G* CSP is further exemplified by the CSP blind tests, which have been running periodically since 1999 as a benchmark for the performance of modern CSP methods. The first test contained four small molecules (two fully rigid, one with a hydroxy group, and one with two rotatable bonds), all in *Z*′ = 1, *G* = 1.^6^ It was not until the third blind test in 2005 that *Z*′ = 2, *G* = 2 crystal structures were introduced.^7^ The fourth blind test in 2009 introduced the first cocrystal, in which *Z*′ ≠ *G*.^8^ The sixth blind test in 2016 introduced the first *G* = 3 crystal structure, though one of the species was Cl^–^ with no rotational degrees of freedom.^9^ The seventh blind test in 2024 stepped up the difficulty with another *G* = 3 crystal structure, but each molecule had 3 rotational degrees of freedom and added degrees of freedom from rotatable bonds.^10^ Of the 19 groups who attempted to predict this crystal, only 1 succeeded, in part because *Z*′ was not made known from the outset of the test, and only 7 of the groups attempted CSP at *Z*′ = 3.

Unfortunately, while uncommon, *Z*′ ≥ 1 crystal structures are not rare. In an analysis of the CCDC’s Crystal Structure Database (CSD), Waddell showed that 10% of crystal structures in the CSD^11^ are *Z*′ ≥ 1.^12^ Furthermore, there are also some multicomponent crystals which are inherently *G* ≥ 1, such as organic frameworks,^13,14^ molecular salts,^13,15^ and cocrystals^16–18^ which can be used to modulate the properties of pharmaceutical materials.^19–21^ Improvement of methods for high-*G* CSP, therefore, is important for the development of generally applicable CSP.

The most common approach to CSP, as evidenced by the seventh blind test,^10^ is quasi- or pseudo-Random Structure Searching (RSS), which consists of (quasi/pseudo) random generation of initial candidate crystal structures, followed by iterative geometry optimization of atomic forces.^22,23^ Many implementations of RSS enforce a minimum interatomic distance within the initial candidate crystal structure as many models (such as Buckingham potentials^24^ and pseudopotential approximations in DFT^25^) perform catastrophically badly at such distances. It is challenging to avoid generating these crystal structures entirely, so they may be discarded after generation or the unit cell volume may be expanded to separate atoms to a safe distance.^22^ The prior approach is simple to implement but potentially yields valid crystals very infrequently and is therefore slow, while the latter biases the crystal structures to large volumes which can lead to long minimization times or failed minimizations. As *G* increases, the likelihood of generating a crystal structure containing unphysically short interatomic distances increases, which slows the rate of the generation of valid candidate crystal structures. CSP is also slowed at the geometry optimization step. An increase in degrees of freedom is often associated with a rougher PES, and therefore more geometry optimization iterations are required to reach a minimum. The increase in cost of the geometry optimization process is further exacerbated by the increase in cost of energy and force evaluation, as all energy models scale with the number of atoms in the system. The final contributing factor to the increased cost is the need to generate and optimize more crystal structures due to the increased number of minima on the PES.

We show that for molecular systems, we can increase the success rate of random structure generation, reduce the number of geometry optimization iterations required per crystal structure, and reduce the number of crystal structures needed to be generated and optimized. This is achieved by performing a short, coarsely sampled CSP with a traditional QRSS method, extracting groups of molecules from the crystal structure as a rigid cluster, and quasi-randomly generating subsequent crystal structures from these clusters rather than a set of independent molecules. We refer to this method as sensible asymmetric units for crystal exploration (SAUCE).

## 2 Methods

### 2.1 Quasi-Random Structure Generation

We assess the efficacy of the proposed methods against a baseline of an established and well-tested crystal structure generator, as implemented in mol-CSPy (known as GLEE) and described in Case et al.’s 2016 paper.^22^ In its latest implementation, the crystal landscape generator begins by converting an integer seed to a quasi-random vector. That vector is then converted to a set of lattice vectors, which are constrained by the crystal’s space group and an acceptable volume range. For each molecule or ion in the asymmetric unit, the quasi-random vector is used to generate a translation (with respect to the origin) and a rotation (with respect to an arbitrary input orientation). At this point, the space group’s symmetry operators are applied to generate the positions of all atoms in the unit cell. All intermolecular distances are then calculated and, if any are shorter than an acceptable tolerance, each cell length is subsequently increased by 1 Å until any collisions are resolved. To avoid overly large cells, each cell length is then iteratively reduced by 1 Å until any further reduction in that cell length would cause a collision. It is essential that as the cell dimensions change, the relative positions of atoms within any given molecule are fixed to avoid distortion of the molecule. Henceforth, any mention of QRSS refers to this method, as opposed to the SAUCE method that we introduce here.

### 2.2 Sensible Asymmetric Units for Crystal Exploration (SAUCE)

In the method described above, each crystal structure’s asymmetric unit is generated purely quasi-randomly. Where there are several independent molecules (high-*G*), these conditions favor the generation of vacuous cells where each molecule may be spaced distantly in order to remediate any intermolecular clashes. Furthermore, the method is entirely chemically agnostic, determining initial coordinates based on geometry, rather than energy. We remedy these problems by building crystals from asymmetric units that are known to be chemically “sensible”, where none of the intermolecular distances are shorter than our tolerances and each molecule is in an energetically favorable environment. This modification slots seamlessly into our existing infrastructure for crystal generation by treating the entire asymmetric unit of *G* molecules as a rigid body. As we would for a molecule or ion, we determine the translation and rotation of that rigid body from a quasi-random vector, and then expand and contract cell lengths to mitigate any clashes. Because the entire asymmetric unit is treated as a rigid body, the relative positions of the molecules within the asymmetric unit do not change as the cell dimensions change; this ensures that the energetically favorable environments are maintained throughout the crystal generation process, and helps to avoid vacuous unit cells as clashes are only possible *between* asymmetric units or periodic images. Note that the asymmetric unit is only treated as rigid during crystal generation; all molecules may move freely and independently during subsequent geometry optimization.

A single asymmetric unit does not facilitate the sampling of all relevant energy minima, and so it is essential to assemble a broad database of asymmetric units to sample. For each generated crystal, we pseudorandomly select an asymmetric unit weighted by the sum of two weighting schemes based on the Boltzmann-like factor,

2 $$W_{j}=\exp{\left(\frac{\epsilon_{j}-\epsilon_{i}}{K}\right)}^{-1}$$

where *W*_*j*_ is the weighting, ϵ_*j*_ is the weighted variable of asymmetric unit *j*, ϵ_*i*_ is the weighted variable of the lowest weighted variable asymmetric unit in the database, and *K* is a constant.

The weighted variable for the first weighting scheme is the utilization of the asymmetric unit, i.e., how many times we have attempted to generate a crystal structure with that asymmetric unit. This scheme exists to avoid overutilization of any particular asymmetric unit, which would otherwise limit the breadth of the search when generating a large number of crystal structures from a small number of asymmetric units, or when a subset of asymmetric units have particularly small values for the second weighted variable. For this weighted variable, we set *K* such that an asymmetric unit with zero use is twice as highly weighted as one with 100 uses. We may amend our preference for the value of *K* as we build experience with the SAUCE algorithm. The best value may depend on the degrees of freedom in the system, but we expect that it is unlikely to have a significant impact, so long as it falls within a sensible range.

The second weighted variable is the energy of the asymmetric unit. This energy is calculated in a vacuum, as if it were a cluster, and with the same energy model that will be used for geometry optimization of generated crystal structures. For this weighted variable, we set *K* to *k*_B_ *T*, where *k*_B_ is the Boltzmann constant, and *T*, the temperature, is selected such that the median energy asymmetric unit has a weight of 0.5 times that of the lowest-energy asymmetric unit. By fitting the temperature to the data set, the weighting distribution is regularized for systems with an arbitrary energy range. In our implementation of SAUCE, we allow for the user to set the energy parameter, *K*, arbitrarily; we recommend between 0.25 and 0.75 for a diverse search with a bias toward low-energy asymmetric units. Our testing of a range of values found that 0.5 served its intended purpose. Determining the optimal value requires further testing and may be dependent on the system and the use case.

We explore two methods for sourcing asymmetric units from geometry-optimized crystals: asymmetric unit transplant (AUT) and unit cell to asymmetric unit (UC2AU).

After determination of the asymmetric unit for the new crystal, the remainder of the crystal generation proceeds much in line with QRSS. Symmetry operations are applied to the asymmetric unit to populate the whole unit cell with molecules. We then adjust the unit cell lengths to relieve intermolecular clashes and minimize cell volume. The key difference between QRSS and SAUCE is that, during the structure generation and initial adjustment of unit cell parameters, QRSS fixes the fractional position of the center of geometry of each molecule and the intramolecular bond lengths. SAUCE fixes the fractional position of the center of geometry of the asymmetric unit and the relative Cartesian positions of all atoms in that asymmetric unit with respect to the center of geometry. This ensures that, as the cell dimensions change, the asymmetric unit remains unchanged. This overall leads to denser, less vacuous cells and a higher success rate at the crystal generation step.

SAUCE is a stochastic structure searching method that biases searches toward low energy structures by fixing some known “sensible” geometric features and (quasi) randomly modifying others. To this end, it is related to related to other Monte Carlo approaches such as basin hopping^26,27^ and also the FUSE approach^28^ for inorganic structure prediction. Modern CSP workflows frequently comprise multiple geometry optimization rounds, with earlier rounds taking advantage of “fast” but “approximate” energy models (such as Buckingham potentials^24^) to quickly approach the local minima, and later rounds using “slow” but “accurate” energy models (such as DFT^25^) to achieve accurate energy rankings.^10^ SAUCE can be applied with any relevant energy model, but only reduces the cost of the first geometry optimization round.

#### 2.2.1 Asymmetric Unit Transplant (AUT)

Our simplest approach to SAUCE is to transplant an asymmetric unit from a geometry-optimized crystal structure to a new unit cell with the same space group. We achieve this by first generating *N* crystal structures with QRSS and geometry optimizing them, where *N* is a user-defined variable but is set to 1000 for all test cases reported in this work. For most cells where *G* > 1, there are multiple ways to represent the asymmetric unit that are equally valid. However, this degeneracy is broken when it is transplanted to a new crystallographic reference frame. We sample a set of asymmetric unit representations by iterating over each molecule in the asymmetric unit and, starting from that molecule, identifying a nearest-neighbor chain of the symmetry-independent molecules (of which there are *G* many). Each of these chains is extracted from the cell as if it were a cluster, and we calculate the energy of that cluster using the same model that is used for geometry optimization. We keep only the lowest-energy cluster and discard the others. We then assembled a database of *N* clusters: one from each crystal structure.

To generate crystal structures with AUT, lattice parameters are selected quasi-randomly, as with QRSS. A cluster from the asymmetric unit database is then pseudorandomly selected, weighted by energy and utilization, as defined in eq 2. The translation and rotation of that cluster is then selected quasi-randomly. This offers an advantage in that the relative positions of molecules in the asymmetric unit are taken from a geometry optimized structure, so these molecules are in favorable relative positions. This is a complete determination of the crystal structure’s asymmetric unit and the remainder of the crystal generation proceeds as described above for SAUCE. The procedure is summarized in Figure 1.

**Figure 1:**
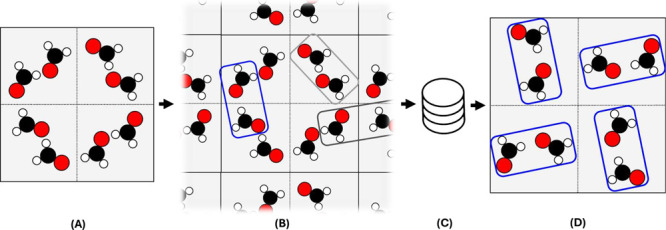
Overview of AUT method for SAUCE. We show a model 2D system where a *Z*′ = 2, *Z* = 8 methanal crystal structure is used to source asymmetric units for a new *Z*′ = 2, *Z* = 8 methanal crystal. (A) Crystal structures are generated by established RSS methods and geometry optimized. (B) Different representations of the asymmetric unit are sampled from a supercell of the optimized crystal. Each representation is treated as a cluster and their energies are calculated using the same model used for the crystal geometry optimizations. (C) The lowest energy cluster from each crystal structure is collated in a database. (D) New crystal structures are generated from the clusters. The cluster is chosen randomly (Boltzmann weighted by energy and utilization), the lattice parameters are chosen quasi-randomly, and the rotation and placement of the cluster in the asymmetric unit are chosen quasi-randomly.

#### 2.2.2 Unit Cell to Asymmetric Unit (UC2AU)

We next propose a method which is applicable only where *Z*′ > 1. Though pseudosymmetry appears often in the asymmetric unit of high-*Z*′ structures,^2,12^ pseudosymmetry occurs only very rarely (before geometry optimization) with QRSS. However, SAUCE offers a path to ensure it by setting the asymmetric unit to the optimized unit cell of a lower *Z*′ crystal. In the UC2AU method, *Z*′ = 1 CSP is performed for space groups which contain the same number of symmetry operators as the target *Z*′. At the end of the CSP, duplicate crystal structures are removed, and for each remaining crystal, all molecules from the unit cell are extracted as a cluster, their energies are calculated, and they are stored in a database. Unlike AUT, the asymmetric units applied in UC2AU have enforced symmetry (see Figure 3). The remainder of the method proceeds as with AUT, with asymmetric units being randomly selected, quasi-randomly placed in quasi-randomly determined lattice parameters, and unit cell lengths optimized. The procedure is summarized in Figure 2.

**Figure 2:**
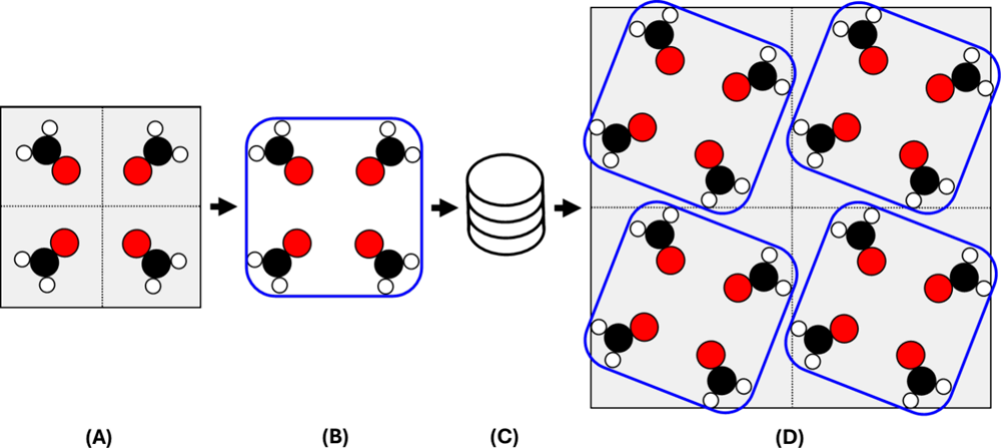
Overview of UC2AU method for SAUCE. We show a model 2D system where a *Z*′ = 1, *Z* = 4 methanal crystal is used to source asymmetric units for a new *Z*′ = 4, *Z* = 16 methanal crystal. (A) Crystals are generated by established RSS methods and geometry optimized. (B) The molecules from the unit cell are extracted as a cluster and its energy calculated using the same model used for the crystal geometry optimizations. (C) A cluster from each crystal structure is collated in a database. (D) New crystal structures are generated from the clusters. The cluster is chosen randomly (Boltzmann weighted by energy and utilization), the lattice parameters are chosen quasi-randomly, and the rotation and placement of the cluster in the asymmetric unit are chosen quasi-randomly.

**Figure 3:**
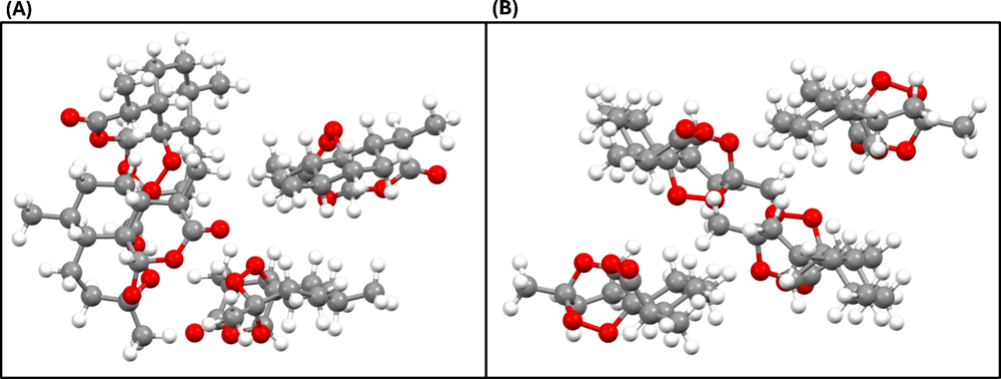
Lowest energy asymmetric units generated from AUT (A) and UC2AU (B) for CSP of artemisinin in space group *P*1 with *Z*′ = 4. Both asymmetric units were sourced from crystal structures with four molecules in the unit cell, but the crystal structure used for AUT had only one symmetry operator (the identity) and the crystal structure used for UC2AU had four symmetry operators. The difference in the symmetry of the asymmetric units is obvious. Despite this, the energy of cohesion of these asymmetric units differs by only 0.46 kJ/mol.

A high *Z*′ (*Z*′ > 1) CSP is typically preceded by CSP with lower *Z*′ values as these are far cheaper to perform and contribute to a complete description of the landscape. For this reason, we consider there to be zero added cost for sourcing asymmetric units for UC2AU (the cost of extracting molecules from a unit cell is negligible). Therefore, the rates reported for UC2AU in this work do not account for the cost of the initial *Z*′ = 1 CSP. It is standard practice in CSP to sample a subset of space groups which most frequently appear in molecular crystals. For crystals with *Z*′ ≤ 1, 99.4% of crystals are represented by just 26 space groups,^3^ and these space groups contain a range of numbers of symmetry operators.

Even a somewhat minimalist *Z*′ = 1 CSP covering the top 10 space groups provides a diverse set of symmetries for a *Z*′ = 4 CSP and a thorough CSP of the top 26 space groups would provide diverse enough symmetries for *Z*′ = 2. Odd numbered *Z*′ (>1) are somewhat trickier: only one space group with 3 symmetry operators appears in the top 26 space groups, and there are only 3 in the entire list of all space groups. Across all space groups, 5 and 7 never appear, and only one space group has 9 symmetry operators. Fortunately, only 0.7% of all known molecular crystals are *Z*′ ≥ 4.^12^

In this work, we generate only 5000 crystal structures per space group as a source of unit cells for UC2 AU and extract unit cells only from unique crystals within 20 kJ/mol of the global energy minimum, demonstrating that a large data set is not required for UC2AU to be effective.

### 2.3 Geometry Optimizations

All geometry optimizations reported in this work were performed in DMACRYS 2.3.0 with a modified Newton–Raphson optimization scheme.^29^ We employ a two-step optimization with an initial “coarse” minimization with atomic point-charge electrostatics followed by a finer minimization with atomic multipole electrostatics. At both steps, we employ a modified W99rev^30^ Buckingham potential^24^ for dispersion interactions.

### 2.4 Structure Comparison

To measure the number of instances of a particular crystal structure appearing in a database, we perform a two step process of deduplication and structure matching. In the first step, we apply an efficient deduplication procedure of dynamic time-warping (DTW) of simulated powder X-ray diffraction (PXRD) patterns^31^ (computed with PLATON^32^) to an entire database of crystal structures. Tolerances for structure similarity have been tuned such that “obvious” matches are recorded as such, but edge cases are treated as nonmatches. This yields a database of “unique” crystal structures and a list of their duplicate crystals found via CSP. It is computationally more efficient to perform structure matching against this deduplicated database than the entire database.

To the database of unique crystals, we next apply COMPACK,^33^ as implemented in the CSD Python API.^34^ We overlaid 30-molecule clusters, and structures were considered to be a match if the difference in the intermolecular atom–atom distances and angles between the two crystal structures was less than 30% and 30°, respectively. With COMPACK, each unique crystal structure from the CSP is compared to a reference crystal structure, which is the experimentally observed crystal (or CSP global energy minimum if no experimental crystal is reported). It is expected that one or more crystal structure matches are found, but each of these matching structures may also be associated with a number of duplicate crystal structures, as determined by DTW PXRD. The total number of times that the reference structure was discovered is equal to the sum of the matching crystal structures and their duplicate crystal structures.

Importantly, we do not compare CSP structures directly to the experimental crystals reported in the CSD. Each experimental crystal is geometry optimized with the same model used for the CSP. This ensures that structure comparisons are measuring the performance of the structure searching methods rather than the performance of the energy model.

### 2.5 Systems Studied

To test the efficacy of SAUCE, we apply QRSS, AUT, and UC2AU to a diverse set of systems. We have selected the systems to achieve a diversity in *Z*′ and *G*, covering *Z*′ = 1–4 and *G* = 2–5. We also strived for diversity in intermolecular interactions and have therefore selected crystal structures where the dominant forces are van der Waals, hydrogen bonding, or electrostatics. In total, six unique systems are considered, summarized in Figure 4, comprising pure (single-component) crystals and salts. All systems are well understood and have been studied with CSP previously. We include artemisinin in particular because it was used as a reference for QRSS in our previous work.^22^

**Figure 4:**
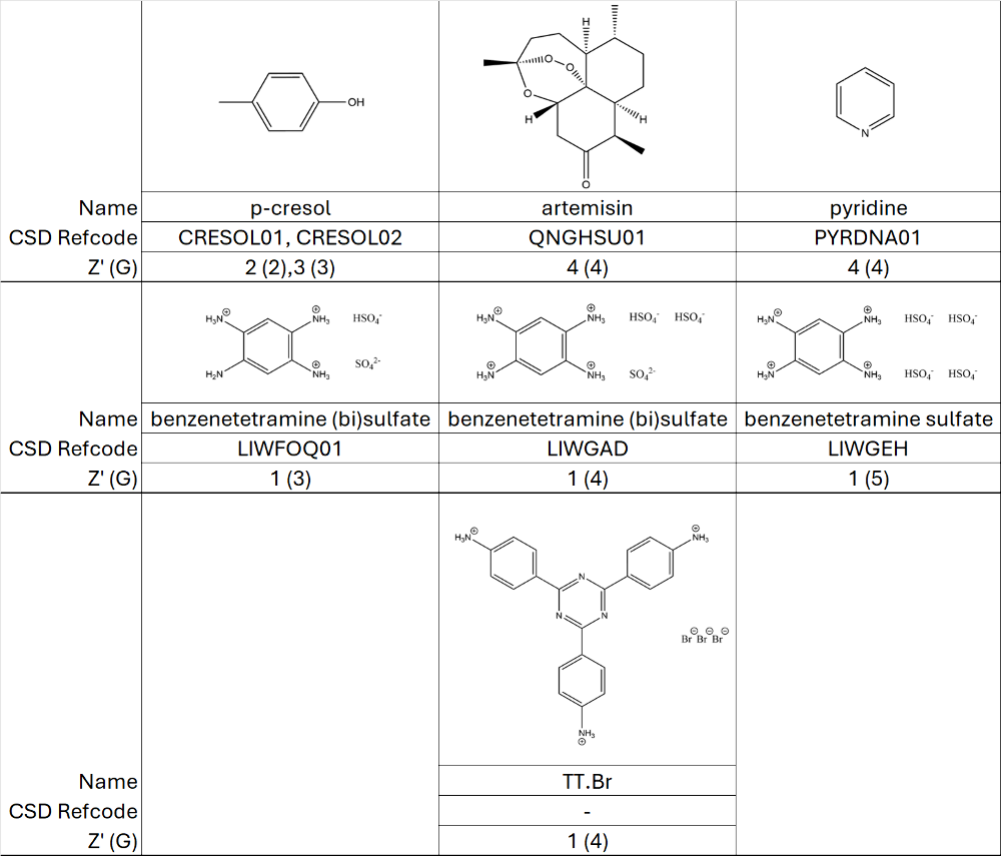
Table of systems tested in this work. Table shows skeletal structure of formula unit, system name, CSD refcode of experimentally observed crystals which are compared against, and *Z*′ of each of these crystal structures.

The planar, aromatic molecule *p*-cresol has a hydrogen bonding acceptor/donor hydroxy group. It has experimentally observed crystals with *Z*′ = 2 in space group *P*2_1_/*c* and with *Z*′ = 3 in space group *C*2/*c*.

Artemisinin is a well-studied nonplanar molecule with hydrogen bond acceptors but no hydrogen bond donors. Intermolecular interactions in the crystal are therefore largely driven by van der Waals forces. Artemisinin has a known *Z*′ = 4 *P*1 crystal structure, making it well suited to assessing SAUCE.

Pyridine is the last of the pure (single-component) systems. Pyridine is a planar molecule, again with a hydrogen bonding acceptor but only weak C–H hydrogen bond donors. Pyridine has a known *Z*′ = 4 *Pna*2_1_ crystal structure.

Benzenetetramines (BTAs) have been observed to form a range of salts with hydrogen sulfates and bisulfates.^35^ We have selected three of these salts, with *G* = 3, 4, and 5. Note that the protonation states of the BTA and the stoichiometries are not the same among the three systems.

4,4’,4”-(1,3,5-triazine-2,4,6-triyl)­tris­[benzenamine] (TT) is a planar ion with charge +3 and hydrogen bonding acceptors and donors. It has been observed to form porous crystal salts when crystallized with halide counterions.^13^ A single crystal structure of TT.Br is not available, but its structure has been determined by comparison of PXRD to the global energy minimum from CSP.^13^ TT.Br is *G* = 4 and the global minimum from CSP is found in space group *C*2/*c*.

## 3 Results

We first seek to establish the extent of the correlation between an asymmetric unit and the geometry optimized crystal structures generated from it. To this end, we have performed five separate CSPs on artemisinin in space group *P*1 with *Z*′ = 4 with the UC2AU method. Each of these CSPs uses a different single asymmetric unit and was performed until 1000 crystal structures were optimized, yielding a total of 5000 crystal structures. Violin plots of the energies and densities of the resultant structures (Figure 5) reveals no apparent correlation between the density or energy of the optimized crystal structure, and the asymmetric unit from which it was generated. It is therefore unlikely that a particular density-energy range can be targetted with SAUCE, but it does imply that a large number of asymmetric units are not required in order to effectively explore the landscape. It should not be inferred that any minimum on the landscape is accessible from any asymmetric unit, as SAUCE with single asymmetric units yield fewer unique crystal structures than QRSS or SAUCE performed with a large set of asymmetric units. The required number of asymmetric units is likely to be correlated with the dimensionality of the energy surface and number of minima on the landscape, and therefore we would expect to require a larger number of asymmetric units where *G* is large. In the analysis of specific systems, we go on to show that 1000 asymmetric units is appropriate for most systems.

**Figure 5:**
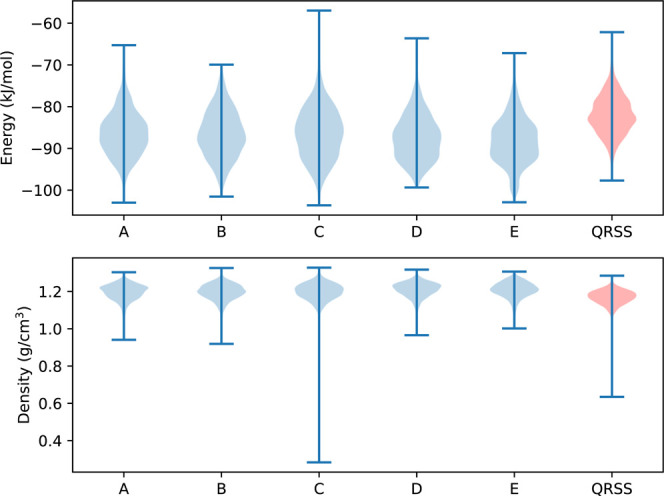
Violin plots contrasting energy and density of *Z*′ = 4 artemisinin crystal structures in space group *P*1. Each violin plot represents 1000 crystal structures generated by UC2AU (blue) and QRSS (red), and then geometry optimized. The UC2AU plots show the output from individual asymmetric units (A, B, C, D, or E). Whiskers show the minimum and maximum value in each data set. UC2AU crystal structures trend toward lower energies and higher densities than QRSS, but crystal structures generated from each asymmetric unit cover a similarly broad distribution of energies and densities. We infer that each UC2AU is capable of generating crystal structures across the full density-energy range from each asymmetric unit.

For the *G* = 3 benzenetetramine (bi)­sulfate in space group *Cc*, we have again performed single asymmetric unit SAUCE, but for this system, we have performed 100 CSPs with AUT, each using a different starting asymmetric unit. In Figure 6a we show that there is a strong correlation between the time required to generate and optimize 1000 crystal structures and the energy of the asymmetric unit. Low-energy asymmetric units tend to result in faster CSP times. We include another density-energy landscape (Figure 6b), colored by the energy of the asymmetric unit, which shows that there is no apparent correlation between the energy of the asymmetric unit and the density or energy of the optimized crystal structure.

**Figure 6:**
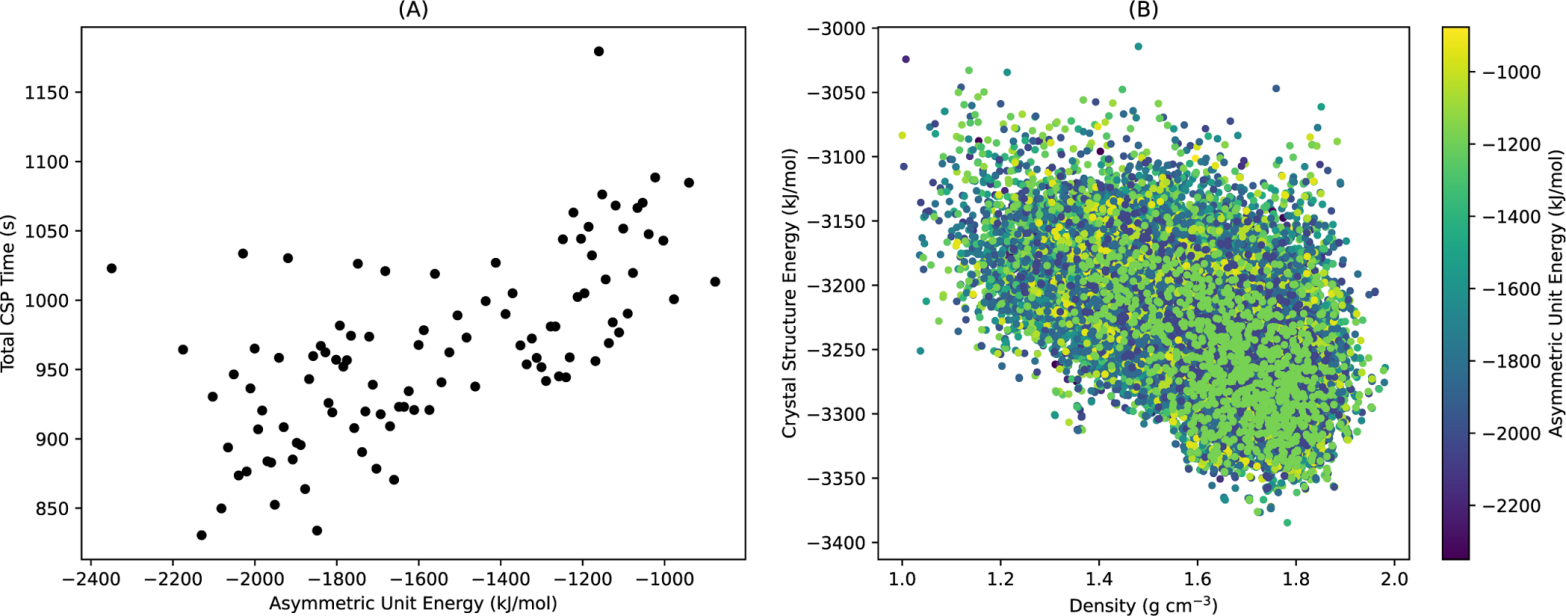
100 CSPs, each containing 1000 crystal structures, for a *G* = 3 benzenetetramine (bis)­sulfate salt. CSP was performed with the AUT method with only a single asymmetric unit each. (A) Energy of asymmetric unit used against total time spent on a 1000-crystal CSP with that asymmetric units. The distribution shows that high energy asymmetric units yield crystals which take longer to optimize. (B) Density-energy crystal landscape, showing that there is no apparent correlation between the energy of the asymmetric unit and the energy or density of the optimized crystal. Data points are colored according to the energy of the asymmetric unit, where purple is low energy and yellow is high energy.

In order to reduce the time spent on CSP, random selection of asymmetric units at the crystal generation step is therefore weighted on the energy of the asymmetric unit, as per eq 2.

We compare the performance of SAUCE to QRSS in five ways. First, we calculate the rate of acquisition of optimized crystal structures by dividing the number of crystal structures generated and optimized by the total time spent on the CSP. This value is hardware dependent, so we report the relative rate of acquisition between the methods. In addition to knowing how quickly crystal structures are acquired, we also want to know whether these crystals are of low energy. We therefore monitor the evolution of the mean energy of the 100 lowest-energy crystal structures over time. The global minimum is not necessarily the only structure of interest; therefore, we want to know how efficiently the overall landscape is sampled. To gain insight into this, we assign each crystal structure from our landscape to a density-energy bin and plot the difference between the occupation of each of these bins between the methods. Areas of the landscape that are explored more by SAUCE than QRSS have a positive value, and areas of the landscape that are explored less by SAUCE than QRSS have a negative value. The end goal of CSP in most cases is to discover an experimentally realizable crystal. For our last assessment, we search the landscapes for matches to the experimentally determined crystal structure and compare the number of hits between SAUCE and QRSS. The relative rate of discovery is not normalized for the speed of acquisition of crystal structures but reflects the relative number of times the experimental crystal is discovered within a given time window.

We summarize our quantitative results as relative rates in Tables 1, 2, and 3 and the absolute values are reported in the Supporting Information.

**Table 1:** Change in Rate of Acquisition of Geometry Optimized Crystal Structures (Crystal Structures Per Second) by AUT and UC2AU with Respect to QRSS^a^

system	*Z*′	*G*	space group	AUT (%)	UC2AU (%)
*p*-cresol	2	2	14	+4	–7
*p*-cresol	3	3	15	+19	+21
artemisinin	4	4	1	+37	+30
pyridine	4	4	33	+12	+9
BTA (bi)sulfate	1	3	9	+31	-
BTA (bi)sulfate	1	4	7 (13)	+22	-
BTA sulfate	1	5	4 (12)	+23	-
TT.Br	1	4	15	+12	-

a Rate for UC2AU is represented with a “-” where the method is not applicable (*Z*′ = 1).

**Table 2:** Change in Frequency of Errors (Errors Per Crystal Structure) During Crystal Generation (Gen) and Geometry Optimization (Opt) by AUT and UC2AU with Respect to QRSS^a^

				AUT		UC2AU	
system	*Z*′	*G*	space group	Gen (%)	Opt (%)	Gen (%)	Opt (%)
*p*-cresol	2	2	14	–60	–22	–60	–22
*p*-cresol	3	3	15	–75	–46	–75	–46
artemisinin	4	4	1	–95	–73	–96	–72
pyridine	4	4	33	–91	–53	–93	–57
BTA (bi)sulfate	1	3	9	–83	–84	-	-
BTA (bi)sulfate	1	4	7 (13)	–91	–66	-	-
BTA sulfate	1	5	4 (12)	–96	–36	-	-
TT.Br	1	4	15	–70	–28	-	-

a Errors during generation are largely attributed to crystal structures exceeding the volume tolerance. Errors during geometry optimization are largely attributed to a failure to converge and Buckingham catastrophes. Rate for UC2 AU is represented with a “-”, where the method is not applicable (*Z*′ = 1).

**Table 3:** Change in the Rate of Discovery (Crystal Structures per Second) of Experimentally Observed (or Global Minimum if None is Available) Crystal by AUT and UC2AU with Respect to QRSS^a^

system	*Z*′	*G*	space group	AUT (%)	UC2AU (%)
*p*-cresol	2	2	14	–4	+20
*p*-cresol	3	3	15	+100	–100
artemisinin	4	4	1	+210	+340
pyridine	4	4	33	+280	+400
BTA (bi)sulfate	1	3	9	+1710	-
BTA (bi)sulfate	1	4	7 (13)	+670	-
BTA sulfate	1	5	4 (12)	+10	-
TT.Br	1	4	15	+600	-

a Rate for UC2AU is represented with a “-”, where the method is not applicable (*Z*′ = 1).

### 3.1 *p*-Cresol

We have performed CSP with QRSS, AUT and UC2AU for a fixed period of time in the appropriate *Z*′ and space group combinations to reproduce the known crystal structures. For UC2AU, we sourced asymmetric units from *Z*′ = 1 CSPs in space groups with two molecules in the unit cell: P1̅ , *P*2_1_, and Pc. For the *Z*′ = 2 crystal structure, we find that the performance of SAUCE is comparable to that of QRSS. AUT yielded 4% more crystals and UC2AU yielded 7% fewer crystals than QRSS over the course of the CSP. This is the only instance in our experimentation where SAUCE yields crystal structures slower than QRSS. We supplement these results by plotting the evolution of the mean energy of the 100 lowest energy crystal structures against time spent on CSP (Figure 7). While AUT and UC2AU are comparable for the duration of the run, QRSS discovers low energy crystal structures more frequently. Finally, we searched the output databases for matches to the experimentally observed crystals and find that while AUT finds the experimentally observed crystal structure 4% slower than QRSS, UC2AU finds the experimentally observed crystal structure 20% faster.

**Figure 7:**
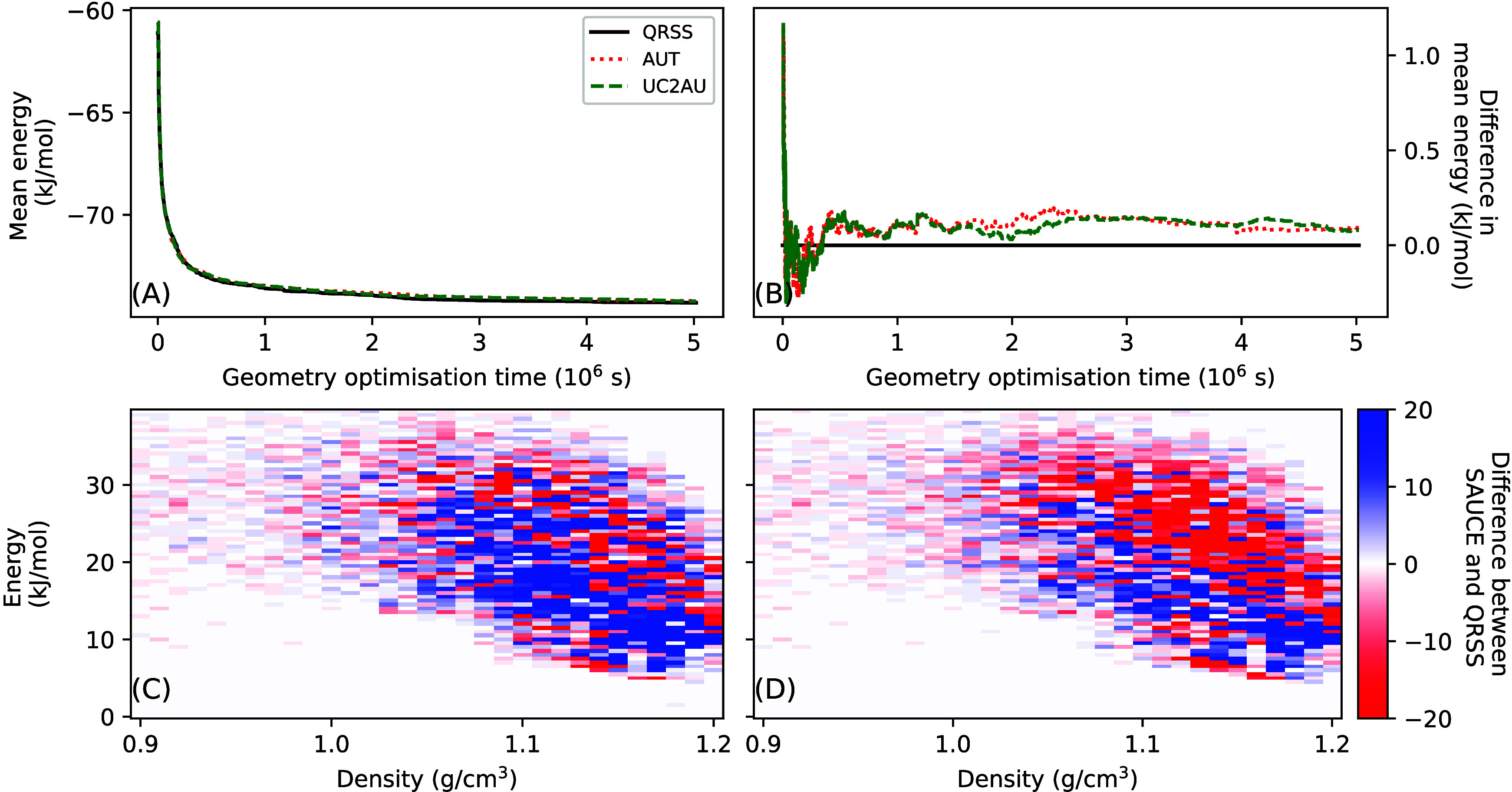
Performance of AUT and UC2AU against QRSS for *Z*′ = 2 *P*2_1_/*c* *p*-cresol. Top shows the evolution of the mean energy of the 100 lowest energy crystal structures of low energy crystal structures over the course of a CSP. The first 150 are truncated. The *x*-axis shows cumulative time spent on valid geometry optimizations and the *y*-axis shows (A) the absolute mean energy and (B) the difference from QRSS. Black: QRSS; Red: AUT; Green: UC2AU. Bottom shows the difference between two *Z*′ = 2, *P*2_1_/*c* *p*-cresol landscapes generated by QRSS and AUT (C), and QRSS and UC2AU (D). Each landscape is binned by density and energy, and the QRSS landscape is subtracted from the SAUCE landscape. Positive (blue) values indicate bins where SAUCE found more structures than QRSS and negative (red) values indicate bins where SAUCE found fewer structures than QRSS.

For *Z*′ = 3 UC2AU, we have sourced asymmetric units from *Z*′ = 1 CSPs in space groups *P*3, *P*3_1_, and *P*3_2_. By the metric of crystal structures per second, SAUCE performed considerably better than QRSS for *Z*′ = 3 *C*2/*c* *p*-cresol, with AUT and UC2AU respectively showing a 19% and 21% speed-up compared to QRSS. However, the evolution of the mean 100 lowest energies (Figure 8) was similar to *Z*′ = 2 *P*2_1_/*c* *p*-cresol. AUT initially converged at the same rate as QRSS but begin to flatten out at 0.8 × 10^6^ s.

**Figure 8:**
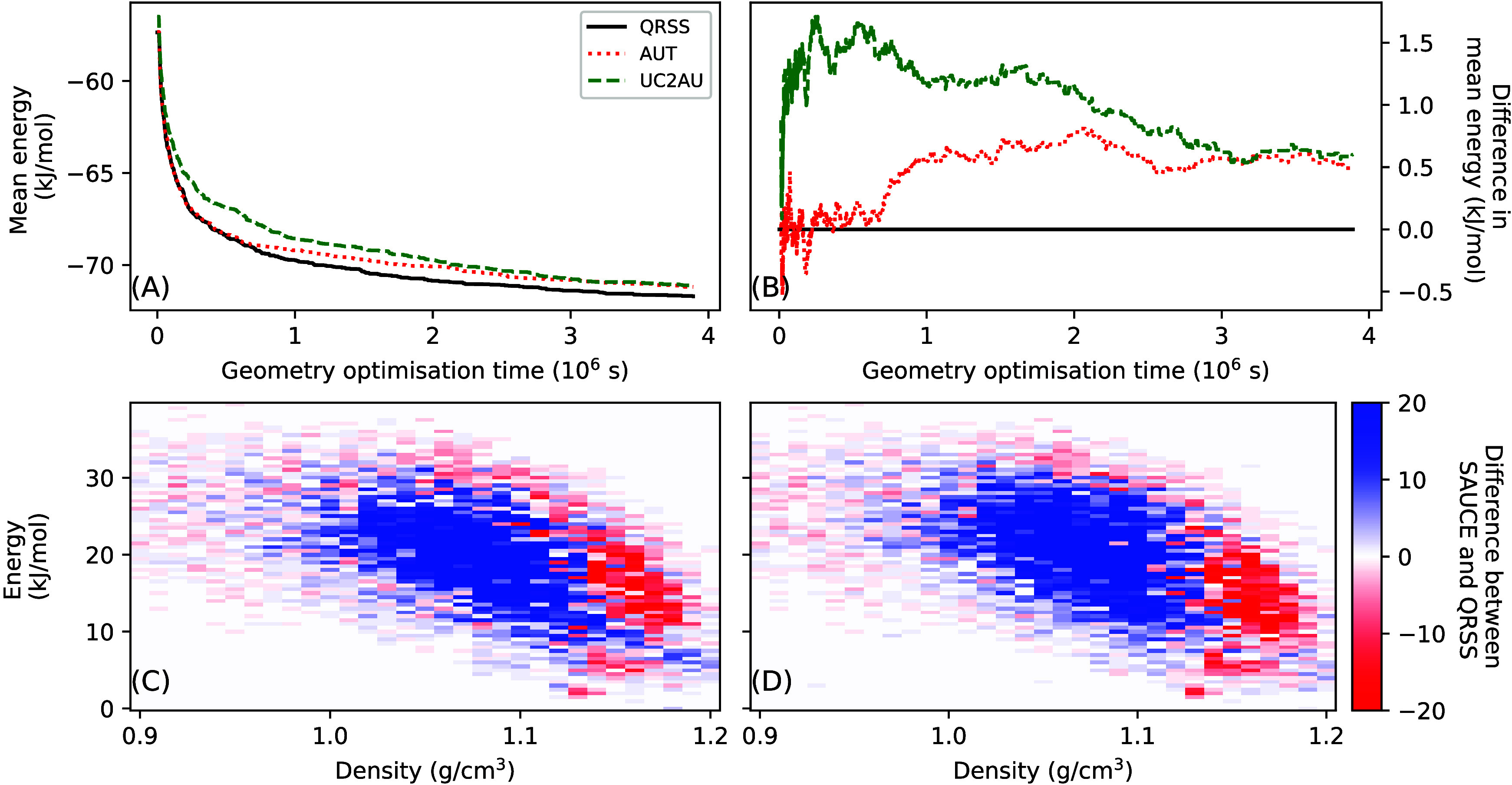
Performance of AUT and UC2AU against QRSS for *Z*′ = 3 *C*2/*c* *p*-cresol. Top shows the evolution of the mean energy of the 100 lowest energy crystal structures of low energy crystal structures over the course of a CSP. The first 150 are truncated. The *x*-axis shows cumulative time spent on valid geometry optimizations and the *y*-axis shows (A) the absolute mean energy and (B) the difference from QRSS. Black: QRSS; Red: AUT; Green: UC2AU. Bottom shows the difference between two *Z*′ = 3 *C*2/*c* *p*-cresol landscapes generated by QRSS and AUT (C) and QRSS and UC2AU (D). Each landscape is binned by density and energy, and the QRSS landscape is subtracted from the SAUCE landscape. Positive (blue) values indicate bins where SAUCE found more structures than QRSS and negative (red) values indicate bins where SAUCE found fewer structures than QRSS.

Determining the relative rate of discovery of the experimental crystal structure posed a challenge for the *Z*′ = 3 target structure. The system had a particularly large number of minima on the landscape which made it difficult to converge. After generating approximately 750000 crystal structures from the same pool of 1000 asymmetric units, we found that 90% of the crystals in the database had only been found once. Space group *C*2/*c* is known to be challenging, in part due to the large number of symmetry operators (8) which leads to a large number of molecules in the unit cell (24 when *G* = 3). For reasons that are not well understood, geometry optimizations are also difficult to converge. *Z*′ = 3 *C*2/*c* *p*-cresol ran for 3× the length of time of the other test sets (except for *G* = 5 benzenetetramine sulfate) and in this time window, QRSS only discovered the experimental crystal structure once, AUT discovered it twice, and UC2AU did not discover it at all. This number of hits is not large enough that we can confidently comment on the relative rate of discovery between the three methods for this system.

For both *Z*′ = 2 *P*2_1_/*c* *p*-cresol and *Z*′ = 3 *C*2/*c* *p*-cresol, it is found that SAUCE finds crystal structures more frequently on the landscape’s leading edge (the low energy edge of the density-energy distribution) and finds crystal structures less frequently on the landscape’s far edge (the high energy edge), as shown in Figures 7 and 8. This shows that crystal structures generated by SAUCE have a greater preference than QRSS for optimizing to leading edge minima than far edge minima. This is a desirable outcome as stable crystal structures are observed to more likely appear on the leading edge.^36^

### 3.2 Artemisinin

We have performed CSP with QRSS, AUT and UC2AU for a fixed period of time in the appropriate *Z*′ and space groups to reproduce the known *Z*′ = 4 crystal structure of artemisinin. For UC2AU, we sourced asymmetric units from *Z*′ = 1 CSPs in space groups: *C*2, *Cc*, *P*2_1_/*c*, *P*2_1_2_1_2_1_, *Pca*2_1_, and *Pna*2_1_.

AUT and UC2AU show a considerable speed-up over QRSS, respectively delivering crystal structures 37% and 30% faster. The AUT evolution of the mean 100 lowest energies (Figure 9) shows a marginal improvement over QRSS, and both QRSS and AUT are significantly outperformed by UC2AU, which shows a large bias toward low energy crystals. Lastly, AUT and UC2AU discover the experimental artemisinin *Z*′ = 4 crystal structure 210% and 340% faster than QRSS, respectively.

**Figure 9:**
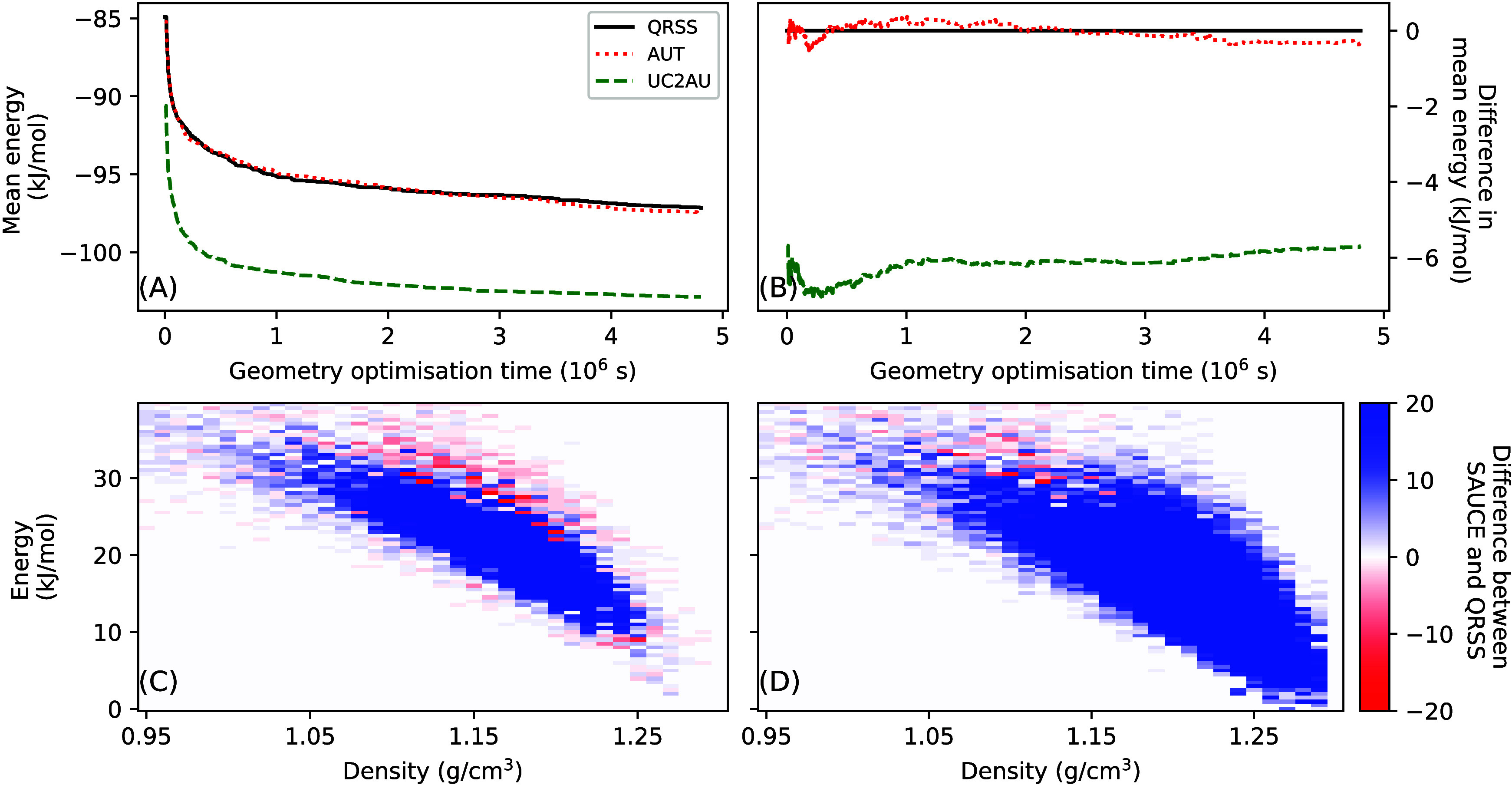
Performance of AUT and UC2AU against QRSS for *Z*′ = 1 *P*1 artemisinin. Top shows the evolution of the mean energy of the 100 lowest energy crystal structures of low energy crystal structures over the course of a CSP. The first 150 are truncated. The *x*-axis shows cumulative time spent on valid geometry optimizations and the *y*-axis shows (A) the absolute mean energy and (B) the difference from QRSS. Black: QRSS; Red: AUT; Green: UC2AU. Bottom shows the difference between two *Z*′ = 1 *P*1 artemisinin landscapes generated by QRSS and AUT (C) and QRSS and UC2AU (D). Each landscape is binned by density and energy, and the QRSS landscape is subtracted from the SAUCE landscape. Positive (blue) values indicate bins where SAUCE found more structures than QRSS and negative (red) values indicate bins where SAUCE found fewer structures than QRSS.

We show in Figure 9 that the speed-up in rate of acquisition for *Z*′ = 4 *P*1 artemisinin is great enough that we explore almost all regions of the landscape more extensively with SAUCE than QRSS. It is also found that UC2AU is more efficient than AUT for acquiring high-density crystal structures, which may be due to the ease of packing an optimized unit cell from another crystal into a *P*1 crystal, and/or pseudosymmetry in the experimental crystal.

Through visual inspection down the *a*-axis, it is apparent that the experimental crystal structure^37^ with CSD^11^ refcode QNGHSU01 displays pseudosymmetry. This was quantified with PLATON’s^32^ ADDSYMM function. Increasing the tolerance of the angle criterium from 1° to 2°, and the distance criterium for coinciding atoms for pseudoinversion symmetry from 0.45 Å to 1 Å, satisfied the criteria for space group *P*1̅, making the crystal *Z*′ = 2. These tolerances are within the range recommended by Brock in her seminal paper on pseudosymmetry in *P*1 crystals.^38^ Thus, asymmetric units from space groups with inversion symmetry may have an advantage in leading to this known crystal structure. Further raising the angle tolerance to 4° satisfied the criteria for space group *P*2_1_/*m*; making the crystal *Z*′ = 1. This space group was not used to source asymmetric units for UC2AU but similar (pseudo) symmetric intermolecular bonding motifs may have been found from crystals in the space groups that were used to source asymmetric units (*C*2, *Cc*, *P*2_1_/*c*, *P*2_1_2_1_2_1_, *Pca*2_1_, and *Pna*2_1_).

### 3.3 Pyridine

We have performed CSP with QRSS, AUT and UC2AU for a fixed period of time in the appropriate *Z*′ and space groups to reproduce the known *Z*′ = 4 crystal structure. For UC2AU, we sourced asymmetric units from *Z*′=1 CSPs in the following space groups with four molecules in the unit cell, *C*2, *Cc*, *P*2_1_/*c*, *P*2_1_2_1_2_1_, *Pca*2_1_, and *Pna*2_1_.

The rate of acquisition of crystal structures for *Z*′ = 4 *Pna*2_1_ pyridine is similar for AUT and UC2AU, which are 12% and 9% faster than QRSS, respectively. The evolution of the mean 100 lowest energies (Figure 10) shows some improvement for both SAUCE methods, with UC2AU slightly outperforming AUT. The experimental crystal structure is discovered 290% faster with AUT and 400% faster with UC2AU compared to QRSS, which is consistent with our results for *Z*′ = 4 *P*1 artemisinin.

**Figure 10:**
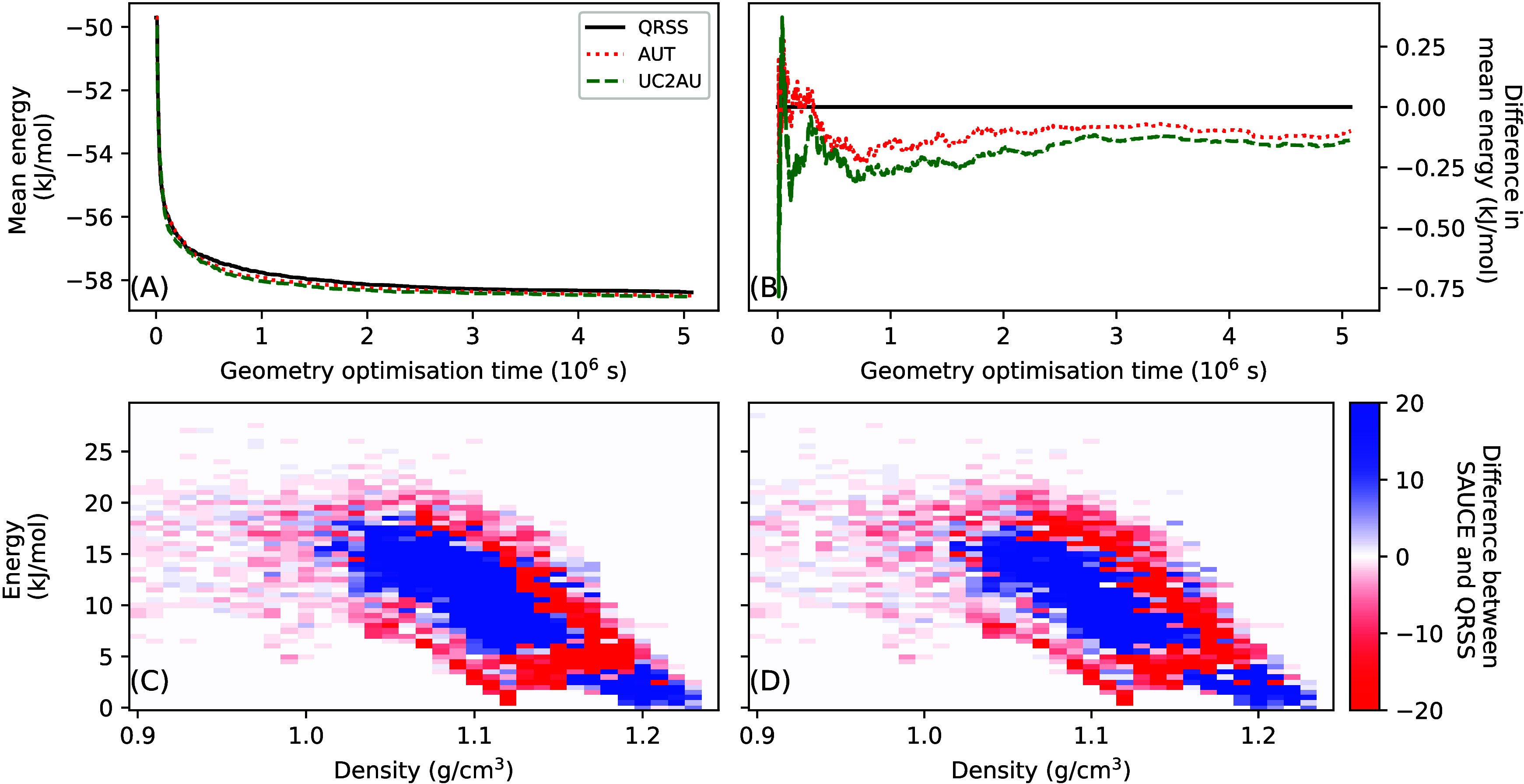
Performance of AUT and UC2AU against QRSS for *Z*′ = 4 *Pna*2_1_ pyridine. Top shows the evolution of the mean energy of the 100 lowest energy crystal structures of low energy crystal structures over the course of a CSP. The first 150 are truncated. The *x*-axis shows cumulative time spent on valid geometry optimizations and the *y*-axis shows (A) the absolute mean energy and (B) the difference from QRSS. Black: QRSS; Red: AUT; Green: UC2AU. Bottom shows the difference between two *Z*′ = 4 *Pna*2_1_ pyridine landscapes generated by QRSS and AUT (C), and QRSS and UC2AU (D). Each landscape is binned by density and energy, and the QRSS landscape is subtracted from the SAUCE landscape. Positive (blue) values indicate bins where SAUCE found more structures than QRSS and negative (red) values indicate bins where SAUCE found fewer structures than QRSS.

*Z*′ = 4 *Pna*2_1_ pyridine crystal structures generated with SAUCE are more likely to be optimized to the leading edge than the far edge, as shown in Figure 10. However, there are regions of the landscape that are less efficiently sampled by AUT and UC2AU; the lower density region of the leading edge of the density-energy distribution and a region between approximately 3–5 kJ/mol above the global energy minimum (see red regions in Figure 10). These regions are explored by SAUCE, but not to the same extent as QRSS. It is possible that this would be mitigated by using a larger, more diverse, database of asymmetric units.

### 3.4 BTA Salts

We next tested SAUCE on salt systems that have multiple, different molecules in their asymmetric units. As these crystal structures are *Z*′ = 1, UC2AU is not possible, but AUT may be applied.

For the three BTA salts, the rate of acquisition of crystal structures is between 22% and 31% faster for AUT than QRSS (Table 1). The evolution of the mean 100 lowest energies (Figures 11, 12, and 13) show small improvements for *G* = 3 and *G* = 4 and more significant improvement for the most complex *G* = 5 system. There is a significant improvement over QRSS in the rate of discovery of the experimental crystal structure for both *G* = 3 and *G* = 4, at 1710% and 670% faster for AUT than QRSS. *G* = 5 showed only a marginal improvement of 10%. To better resolve the difference in performance between QRSS and AUT, we ran the CSPs for twice as long as the other test cases (excluding *Z*′ = 3 *C*2/*c* *p*-cresol). The large difference in performance between *G* = 3 and 4, and *G* = 5 BTA salts is surprising but may be attributed to insufficient sampling of asymmetric units in the later case.

**Figure 11:**
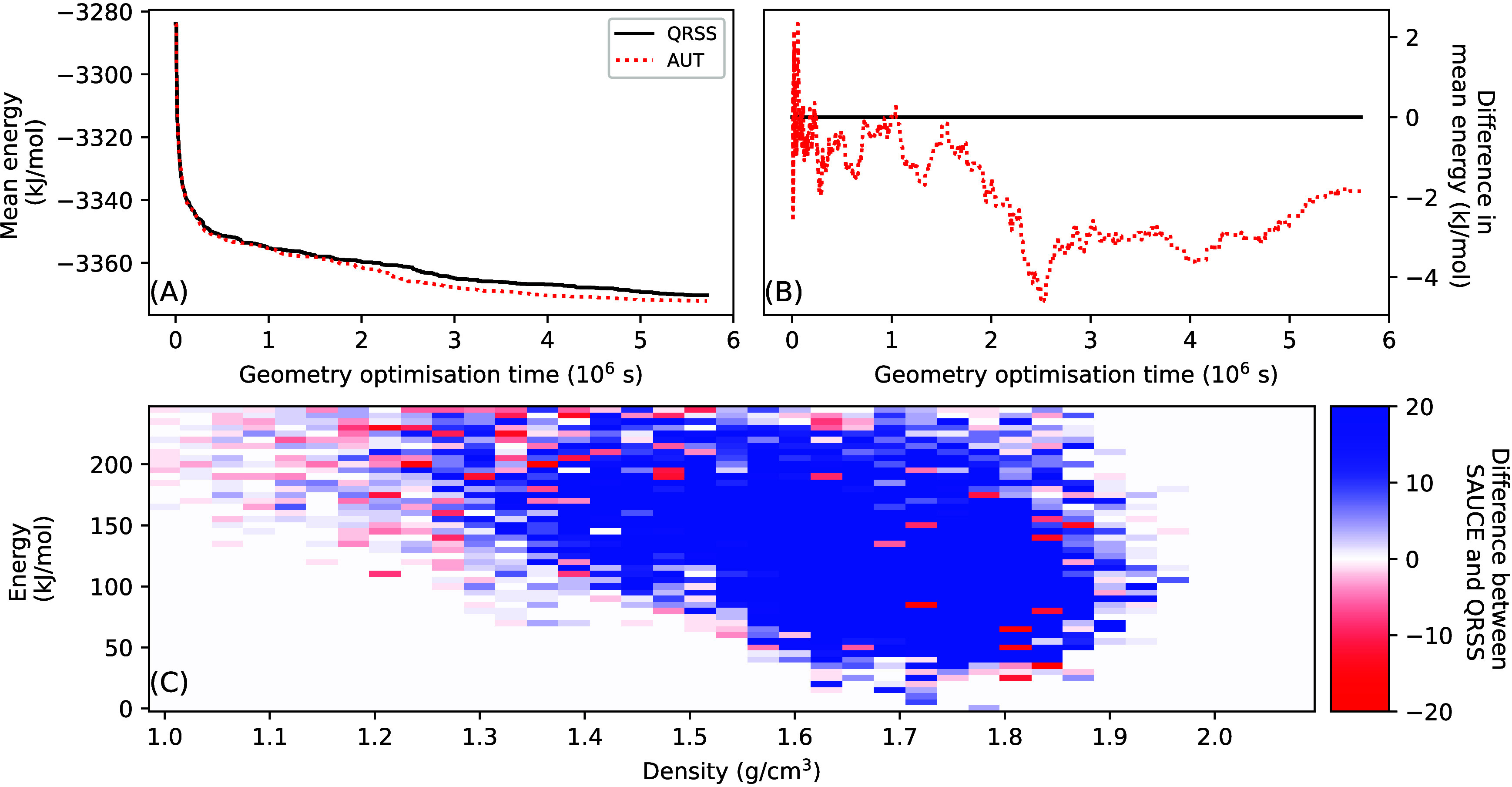
Performance of AUT against QRSS for *G* = 3 *Cc* BTA bis­(sulfate). Top shows the evolution of the mean energy of the 100 lowest energy crystal structures of low energy crystal structures over the course of a CSP. The first 150 are truncated. The *x*-axis shows cumulative time spent on valid geometry optimizations and the *y*-axis shows (A) the absolute mean energy and (B) the difference from QRSS. Black: QRSS; Red: AUT. Bottom shows the difference between two *G* = 3 *Cc* BTA bis­(sulfate) landscapes generated by QRSS and AUT (C). Each landscape is binned by density and energy, and the QRSS landscape is subtracted from the SAUCE landscape. Positive (blue) values indicate bins where SAUCE found more structures than QRSS and negative (red) values indicate bins where SAUCE found fewer structures than QRSS.

**Figure 12:**
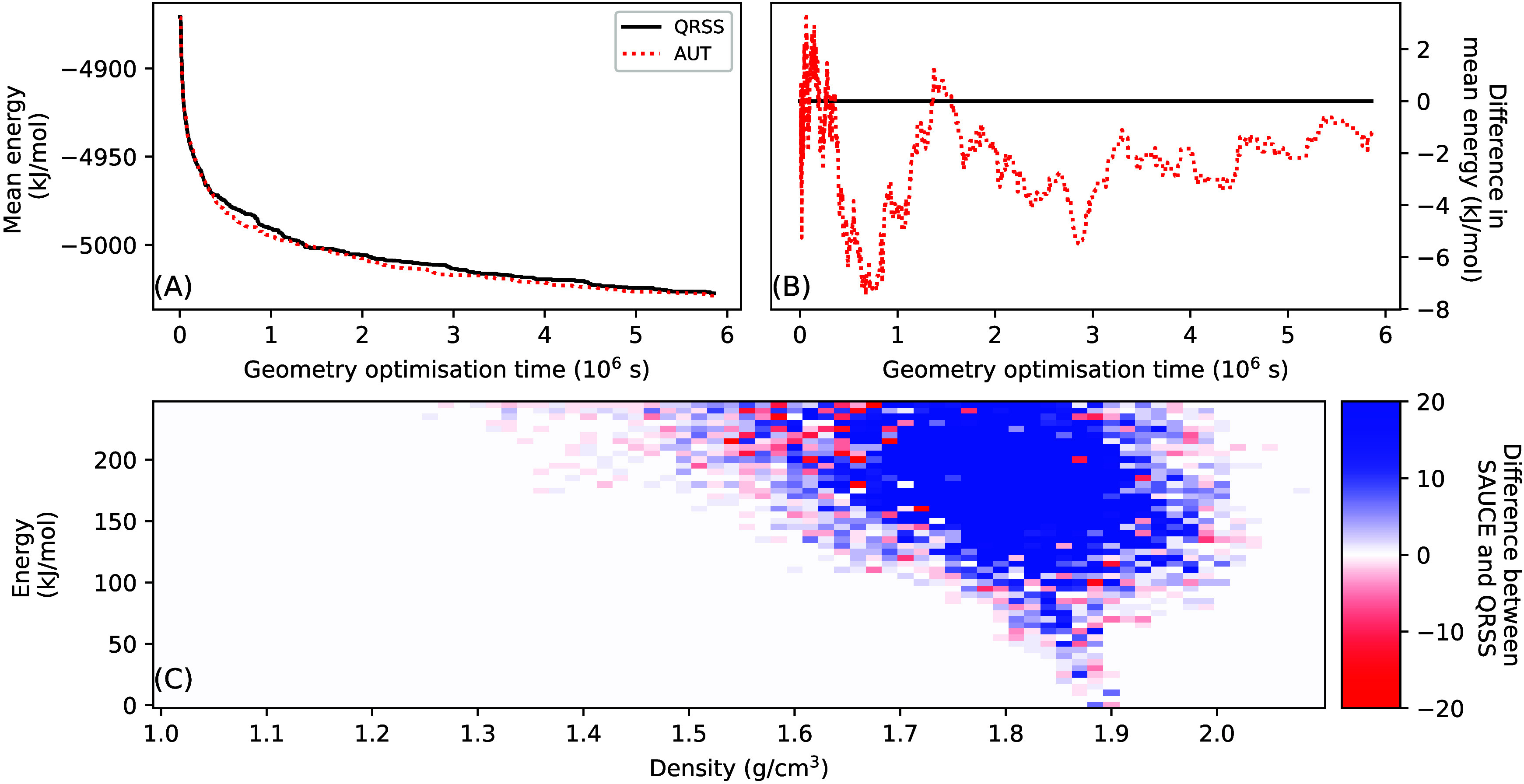
Performance of AUT against QRSS for *G* = 4 Pc BTA bis­(sulfate). Top shows the evolution of the mean energy of the 100 lowest energy crystal structures of low energy crystal structures over the course of a CSP. The first 150 are truncated. The *x*-axis shows cumulative time spent on valid geometry optimizations and the *y*-axis shows (A) the absolute mean energy and (B) the difference from QRSS. Black: QRSS; Red: AUT. Bottom shows the difference between two *G* = 4 Pc BTA bis­(sulfate) landscapes generated by QRSS and AUT (C). Each landscape is binned by density and energy, and the QRSS landscape is subtracted from the SAUCE landscape. Positive (blue) values indicate bins where SAUCE found more structures than QRSS and negative (red) values indicate bins where SAUCE found fewer structures than QRSS.

**Figure 13:**
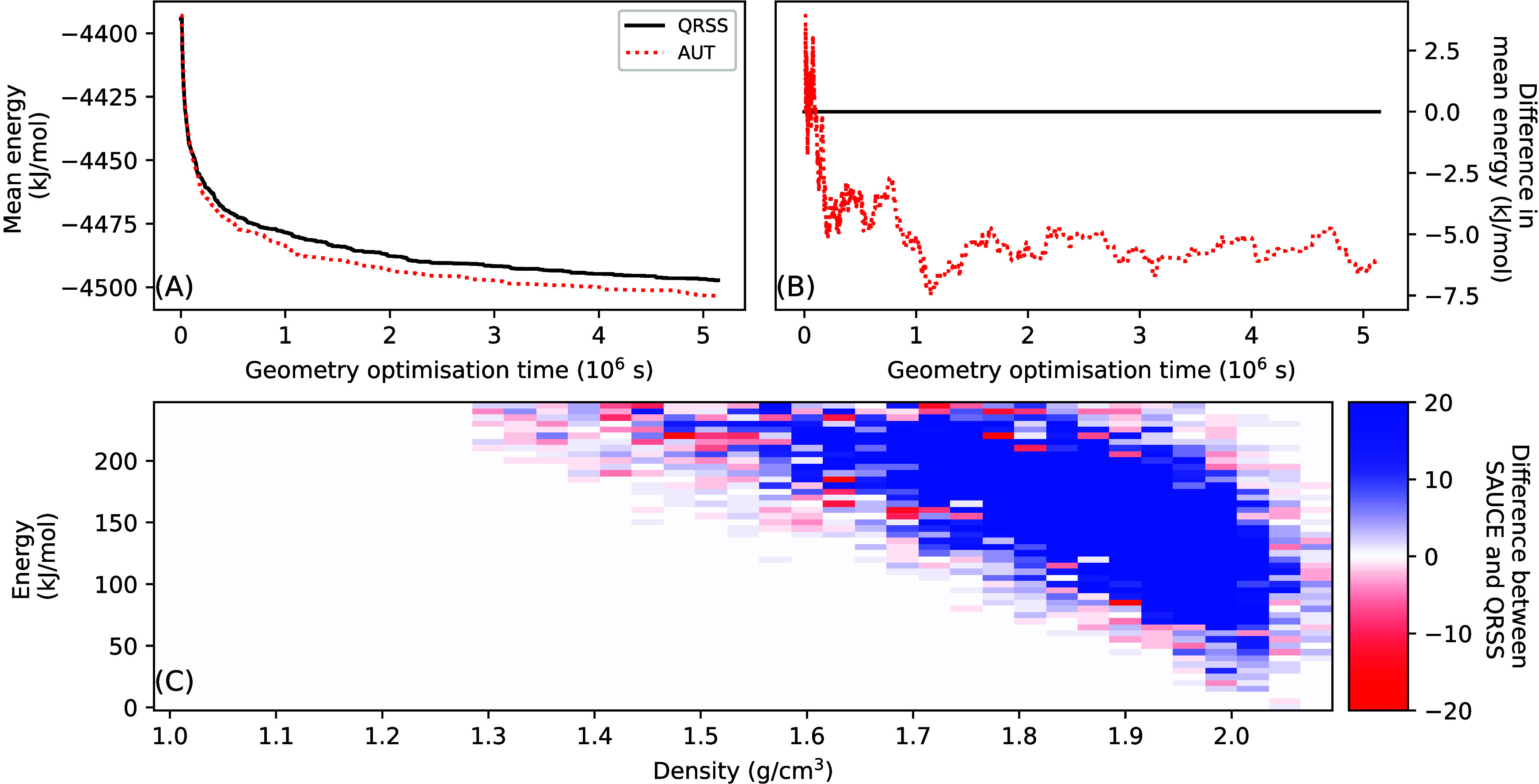
Performance of AUT against QRSS for *G* = 5 *P*2_1_ BTA sulfate. Top shows the evolution of the mean energy of the 100 lowest energy crystal structures of low energy crystal structures over the course of a CSP. The first 150 are truncated. The *x*-axis shows cumulative time spent on valid geometry optimizations and the *y*-axis shows (A) the absolute mean energy and (B) the difference from QRSS. Black: QRSS; Red: AUT. Bottom shows the difference between two *G* = 5 P2_1_ BTA sulfate landscapes generated by QRSS and AUT (C). Each landscape is binned by density and energy, and the QRSS landscape is subtracted from the SAUCE landscape. Positive (blue) values indicate bins where SAUCE found more structures than QRSS and negative (red) values indicate bins where SAUCE found fewer structures than QRSS.

Similar to artemisinin, SAUCE is more efficient than QRSS at exploring all regions of the density-energy distribution for the BTA landscapes (see Figures 11, 12, and 13).

### 3.5 TT.Br

For the final system, TT with 4 Br^–^ counterions, we have found AUT to offer a crystal acquisition speed-up of 12% with respect to QRSS. However, the evolution of the mean 100 lowest energies (14) shows no considerable difference between QRSS and AUT. Despite this, AUT found the global energy minimum, which corresponds to the known porous crystal structure,^13^ 600% more frequently than QRSS.

Discovering the reference structure with either algorithm was much easier for *C*2/*c* TT.Br than for *Z*′ = 3 *C*2/*c* *p*-cresol, despite having one additional free unit. There are two key factors that contribute to this result. Although TT.Br has more free units, Br^–^ has no rotational degrees of freedom, while *p*-cresol does. There are therefore a total of 15 degrees of freedom from the free units in TT.Br and 18 in *p*-cresol. Furthermore, TT.Br has stronger electrostatic interactions which may more easily drive the search to low energy minima.

The landscape of *C*2/*c* TT.Br, Figure 14, is sparse and covers a large density range. SAUCE is effective at generating both high- and low-density crystals, displaying a slight bias for the leading edge over the far edge.

**Figure 14:**
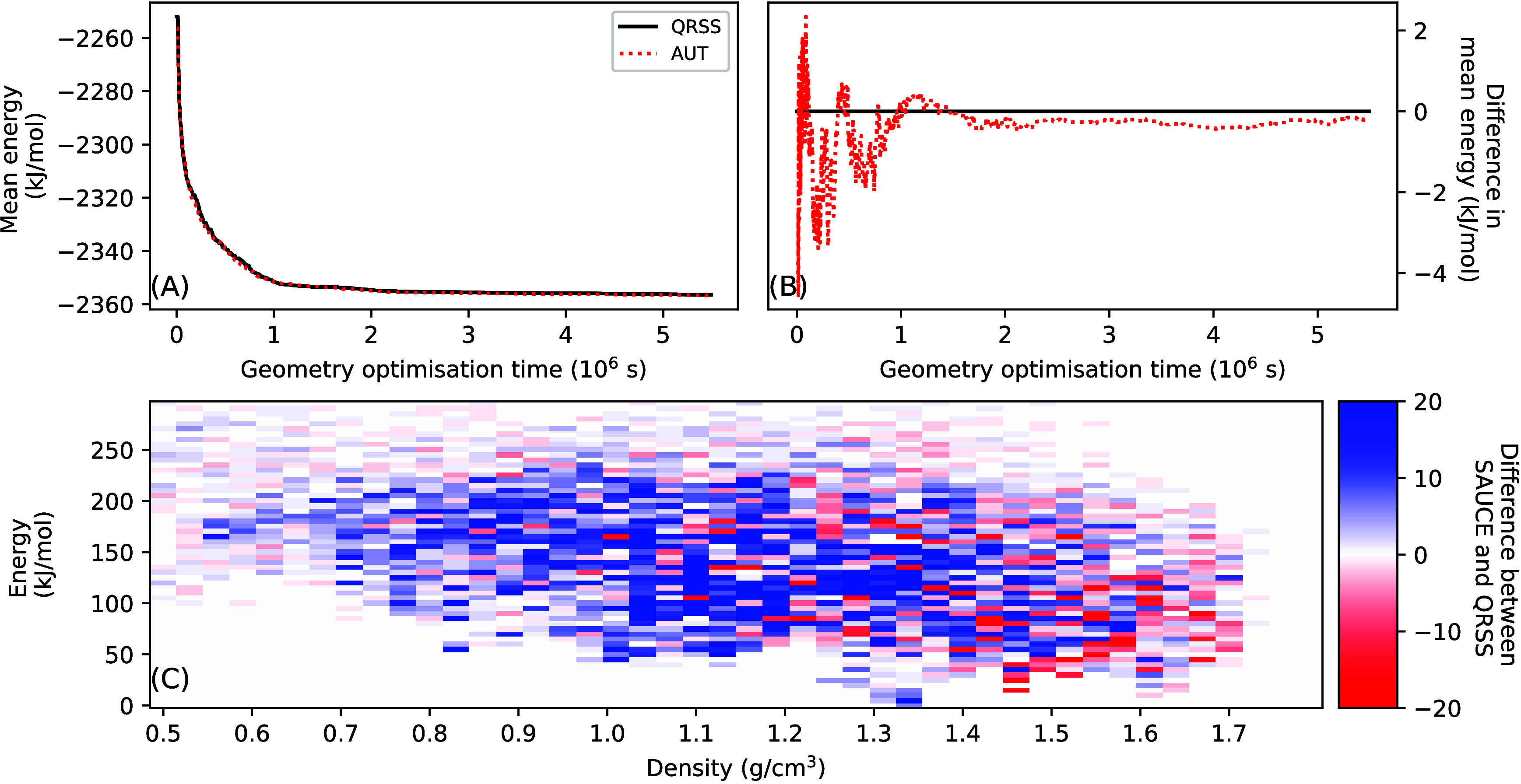
Performance of AUT against QRSS for *C*2/*c* TT.Br. Top shows the evolution of the mean energy of the 100 lowest energy crystal structures of low energy crystal structures over the course of a CSP. The first 150 are truncated. The *x*-axis shows cumulative time spent on valid geometry optimizations and the *y*-axis shows (A) the absolute mean energy and (B) the difference from QRSS. Black: QRSS; Red: AUT. Bottom shows the difference between two *C*2/*c* TT.Br landscapes generated by QRSS and AUT (C). Each landscape is binned by density and energy, and the QRSS landscape is subtracted from the SAUCE landscape. Positive (blue) values indicate bins where SAUCE found more structures than QRSS and negative (red) values indicate bins where SAUCE found fewer structures than QRSS.

### 3.6 Discussion

As a general trend across the systems tested in this work, SAUCE acquires optimized crystal structures faster than QRSS. The speedups from AUT and UC2AU are comparable. These values are summarized in Table 1.

This speed-up can be attributed to two factors. First, the initial, generated crystal structures are closer to local minima, and therefore require fewer geometry optimization iterations to reach the nearest local minimum. Second, as the initial generated crystals are more likely to be “sensible”, the error rate is reduced with respect to QRSS. Table 2 shows SAUCE has between 4% and 30% of the errors that occur during the crystal generation step in QRSS, and between 16% and 78% of the errors that occur during the geometry optimization step. Crystal generation errors occur when intermolecular clashes cannot be resolved by unit cell expansions. Energy minimization errors occur when a time limit or geometry optimization iteration limit is reached, or when Buckingham catastrophes arise. Whenever errors occur, the crystal structure must be discarded and so any time spent on that structure is wasted.

When comparing the evolution of the mean 100 lowest energy crystal structures, the results are mixed. While QRSS outperforms SAUCE for *p*-cresol, in all other cases, SAUCE matches or exceeds the performance of QRSS. A notable example is UC2AU for *Z*′ = 4 *P*1 artemisinin, which converged significantly faster than QRSS and AUT. This may be because the unit cell contained a single asymmetric unit, and so the generated crystal structures are closely related to the crystal structures from which they were sourced.

The greatest improvement between QRSS and SAUCE is in the rate of discovery of the experimental (or global minimum) crystal structures, summarized in Table 3. Testing indicates that a speedup of between 100% and 600% may be typical for SAUCE.

## 4 Conclusions

We have introduced SAUCE; a new method for improving the efficiency of molecular crystal random structure searching for complex systems with multiple independent molecules or ions (*G* > 1). While traditional random structure searching implementations constrain starting structures by geometry alone, SAUCE allows for a more “sensible” selection of asymmetric units which are chemically informed. SAUCE differs from traditional approaches only at the crystal generation step, and can therefore be implemented in established codes with minimal burden.

We have benchmarked the performance of SAUCE against quasi-random structure searching on a range of systems, covering *G* = 1–5, *Z*′ = 1–4, seven different space groups, hydrogen-bonding crystals, van der Waals crystals, molecular salts, and porous molecular crystals. It has been shown that, with SAUCE, CSP is typically 10–30% faster and typically discovers the experimental crystal structure 100–600% more frequently than QRSS. This is achieved because SAUCE is biased toward low energy crystal structures, less frequently generates erroneous structures, and creates the conditions for shorter geometry optimizations. SAUCE is not expected to find crystal structures that cannot be found by QRSS, but rather it finds low energy crystal structures more efficiently. The efficacy of the method is predicated upon building a sufficiently large database of asymmetric units. While the minimum number of required asymmetric units is expected to depend on variables such as *G*, molecular symmetry, and likely bonding motifs, we have shown that for all systems tested in this work, 1000 asymmetric units are sufficient. The cost of acquiring as many as 10000 asymmetric units is a negligible fraction of the total cost of a CSP for most systems and would comfortably accommodate a diverse structure search.

SAUCE is applicable to CSP on any molecular system where *G* exceeds 1 but we find that it has the greatest impact with *G* ≥ 3. SAUCE has already replaced QRSS for such systems in our workflows and we expect the method to make CSP on complex systems more approachable generally.

So far SAUCE has only been benchmarked for rigid molecules but the method is fully transferable to systems featuring flexible molecules. There is no requirement for rigid handling of molecules at the geometry optimization stage. How best to sample molecular conformations at the structure generation stage is still an open question in the CSP community and SAUCE’s ability to inherit “sensible” conformations from crystal structure minima would be advantageous. We will be pursuing this line of research in the near future.

## Supplementary Material



## Data Availability

SAUCE is available via our open-source Python 3 package for molecular CSP, mol-CSPy, on our GitLab: https://gitlab.com/mol-cspy.

## Notes

Number of space group symmetry operators for UC2 AU, absolute numbers of crystal structures generated, absolute numbers of errors, and absolute numbers of discoveries of experimentally observed crystal structures are included (PDF)

## References

[1] van
Eijck B. P., Kroon J. (2000). Structure predictions allowing more
than one molecule in the asymmetric unit. Acta
Crystallogr. B.

[2] Steed K. M., Steed J. W. (2015). Packing Problems: High Zprime Crystal
Structures and
Their Relationship to Cocrystals, Inclusion Compounds, and Polymorphism. Chem. Rev..

[3] Taylor C. R., Butler P. W. V., Day G. M. (2025). Predictive crystallography at scale:
mapping, validating, and learning from 1000 crystal energy landscapes. Faraday Discuss..

[4] Hafizi R., Gittins H., Taylor C. R., Day G. M. (2026). Towards Foundation
Models Trained from Crystal Structure Prediction of the Organic Molecular
Solid State. ChemRxiv.

[5] Maddox J. (1988). Crystals from
first principles. Nature.

[6] Lommerse J. P. M., Motherwell W. D. S., Ammon H. L., Dunitz J. D., Gavezzotti A., Hofmann D. W. M., Leusen F. J. J., Mooij W. T. M., Price S. L., Schweizer B., Schmidt M. U., van Eijck B. P., Verwer P., Williams D. E. (2000). A test of crystal structure prediction
of small organic molecules. Acta Crystallogr.
B.

[7] Day G. M., Motherwell W. D. S., Ammon H. L., Boerrigter S. X. M., Della Valle R. G., Venuti E., Dzyabchenko A., Dunitz J. D., Schweizer B., van Eijck B. P., Erk P., Facelli J. C., Bazterra V. E., Ferraro M. B., Hofmann D. W. M., Leusen F. J. J., Liang C., Pantelides C. C., Karamertzanis P. G., Price S. L., Lewis T. C., Nowell H., Torrisi A., Scheraga H. A., Arnautova Y. A., Schmidt M. U., Verwer P. (2005). A third blind test of crystal structure
prediction. Acta Crystallogr. B.

[8] Day G. M., Cooper T. G., Cruz-Cabeza A. J., Hejczyk K. E., Ammon H. L., Boerrigter S. X. M., Tan J. S., Della Valle R. G., Venuti E., Jose J., Gadre S. R., Desiraju G. R., Thakur T. S., van Eijck B. P., Facelli J. C., Bazterra V. E., Ferraro M. B., Hofmann D. W. M., Neumann M. A., Leusen F. J. J., Kendrick J., Price S. L., Misquitta A. J., Karamertzanis P. G., Welch G. W. A., Scheraga H. A., Arnautova Y. A., Schmidt M. U., van de Streek J., Wolf A. K., Schweizer B. (2009). Significant
progress in predicting the crystal structures of small organic molecules
– a report on the fourth blind test. Acta Crystallogr. B.

[9] Reilly A. M., Cooper R. I., Adjiman C. S., Bhattacharya S., Boese A. D., Brandenburg J. G., Bygrave P. J., Bylsma R., Campbell J. E., Car R., Case D. H., Chadha R., Cole J. C., Cosburn K., Cuppen H. M., Curtis F., Day G. M., DiStasio Jr R. A., Dzyabchenko A., van Eijck B. P., Elking D. M., van den Ende J. A., Facelli J. C., Ferraro M. B., Fusti-Molnar L., Gatsiou C.-A., Gee T. S., de Gelder R., Ghiringhelli L. M., Goto H., Grimme S., Guo R., Hofmann D. W. M., Hoja J., Hylton R. K., Iuzzolino L., Jankiewicz W., de Jong D. T., Kendrick J., de Klerk N. J. J., Ko H.-Y., Kuleshova L. N., Li X., Lohani S., Leusen F. J. J., Lund A. M., Lv J., Ma Y., Marom N., Masunov A. E., McCabe P., McMahon D. P., Meekes H., Metz M. P., Misquitta A. J., Mohamed S., Monserrat B., Needs R. J., Neumann M. A., Nyman J., Obata S., Oberhofer H., Oganov A. R., Orendt A. M., Pagola G. I., Pantelides C. C., Pickard C. J., Podeszwa R., Price L. S., Price S. L., Pulido A., Read M. G., Reuter K., Schneider E., Schober C., Shields G. P., Singh P., Sugden I. J., Szalewicz K., Taylor C. R., Tkatchenko A., Tuckerman M. E., Vacarro F., Vasileiadis M., Vazquez-Mayagoitia A., Vogt L., Wang Y., Watson R. E., de Wijs G. A., Yang J., Zhu Q., Groom C. R. (2016). Report
on the sixth blind test of organic crystal structure prediction methods. Acta Crystallogr. B.

[10] Hunnisett L. M., Nyman J., Francia N., Abraham N. S., Adjiman C. S., Aitipamula S., Alkhidir T., Almehairbi M., Anelli A., Anstine D. M., Anthony J. E., Arnold J. E., Bahrami F., Bellucci M. A., Bhardwaj R. M., Bier I., Bis J. A., Boese A. D., Bowskill D. H., Bramley J., Brandenburg J. G., Braun D. E., Butler P. W. V., Cadden J., Carino S., Chan E. J., Chang C., Cheng B., Clarke S. M., Coles S. J., Cooper R. I., Couch R., Cuadrado R., Darden T., Day G. M., Dietrich H., Ding Y., DiPasquale A., Dhokale B., van Eijck B. P., Elsegood M. R. J., Firaha D., Fu W., Fukuzawa K., Glover J., Goto H., Greenwell C., Guo R., Harter J., Helfferich J., Hofmann D. W. M., Hoja J., Hone J., Hong R., Hutchison G., Ikabata Y., Isayev O., Ishaque O., Jain V., Jin Y., Jing A., Johnson E. R., Jones I., Jose K. V. J., Kabova E. A., Keates A., Kelly P. F., Khakimov D., Konstantinopoulos S., Kuleshova L. N., Li H., Lin X., List A., Liu C., Liu Y. M., Liu Z., Liu Z.-P., Lubach J. W., Marom N., Maryewski A. A., Matsui H., Mattei A., Mayo R. A., Melkumov J. W., Mohamed S., Momenzadeh Abardeh Z., Muddana H. S., Nakayama N., Nayal K. S., Neumann M. A., Nikhar R., Obata S., O'Connor D., Oganov A. R., Okuwaki K., Otero-de-la-Roza A., Pantelides C. C., Parkin S., Pickard C. J., Pilia L., Pivina T., Podeszwa R.ł, Price A. J. A., Price L. S., Price S. L., Probert M. R., Pulido A., Ramteke G. R., Rehman A. U., Reutzel-Edens S. M., Rogal J., Ross M. J., Rumson A. F., Sadiq G., Saeed Z. M., Salimi A., Salvalaglio M., Sanders de Almada L., Sasikumar K., Sekharan S., Shang C., Shankland K., Shinohara K., Shi B., Shi X., Skillman A. G., Song H., Strasser N., van de Streek J., Sugden I. J., Sun G., Szalewicz K., Tan B. I., Tan L., Tarczynski F., Taylor C. R., Tkatchenko A., Tom R., Tuckerman M. E., Utsumi Y., Vogt-Maranto L., Weatherston J., Wilkinson L. J., Willacy R. D., Wojtas L., Woollam G. R., Yang Z., Yonemochi E., Yue X., Zeng Q., Zhang Y., Zhou T., Zhou Y., Zubatyuk R., Cole J. C. (2024). The seventh blind test of crystal structure prediction:
structure generation methods. Acta Crystallogr.
B.

[11] Groom C. R., Bruno I. J., Lightfoot M. P., Ward S. C. (2016). The Cambridge Structural
Database. Acta Crystallogr..

[12] Waddell P. G. (2025). A lot to
unpack: a decade in high Z crystal structures. CrystEngComm.

[13] O’Shaughnessy M., Glover J., Hafizi R., Barhi M., Clowes R., Chong S. Y., Argent S. P., Day G. M., Cooper A. I. (2024). Porous
isoreticular non-metal organic frameworks. Nature.

[14] Xu Y., Marrett J. M., Titi H. M., Darby J. P., Morris A. J., Friščić T., Arhangelskis M. (2023). Experimentally
Validated Ab Initio Crystal Structure Prediction of Novel Metal–Organic
Framework Materials. J. Am. Chem. Soc..

[15] Patel J., Leduc Z., Nunez Avila A. G., Glover J. A., Wu K., Zhang Y., Zhang J., Zhai X., Jing H., Chen A. M., Chartrand D., Maris T., Day G. M., Wuest J. D. (2024). Exploring Polymorphism:
Hydrochloride Salts of Pitolisant
and Analogues. Cryst. Growth Des..

[16] Yang S., Day G. M. (2022). Global analysis
of the energy landscapes of molecular
crystal structures by applying the threshold algorithm. Commun. Chem..

[17] Chan H. C. S., Kendrick J., Neumann M. A., Leusen F. J. J. (2013). Towards ab initio
screening of co-crystal formation through lattice energy calculations
and crystal structure prediction of nicotinamide, isonicotinamide,
picolinamide and paracetamol multi-component crystals. CrystEngComm.

[18] Abramov Y. A., Iuzzolino L., Jin Y., York G., Chen C.-H., Shultz C. S., Yang Z., Chang C., Shi B., Zhou T., Greenwell C., Sekharan S., Lee A. Y. (2023). Cocrystal
Synthesis through Crystal Structure Prediction. Mol. Pharmaceutics.

[19] Bavishi D. D., Borkhataria C. H. (2016). Spring
and parachute: How cocrystals enhance solubility. Prog. Cryst. Growth Ch.

[20] Karki S., Friščić T., Fábián L., Laity P. R., Day G. M., Jones W. (2009). Improving
Mechanical
Properties of Crystalline Solidsby Cocrystal Formation: New Compressible
Forms ofParacetamol. Adv. Mater..

[21] Yan D., Delori A., Lloyd G. O., Friščić T., Day G. M., Jones W., Lu J., Wei M., Evans D. G., Duan X. (2011). A Cocrystal Strategy
to Tune the
Luminescent Properties of Stilbene-Type Organic Solid-State Materials. Angew. Chem. Int. Ed.

[22] Case D. H., Campbell J. E., Bygrave P. J., Day G. M. (2016). Convergence Properties
of Crystal Structure Prediction by Quasi-Random Sampling. J. Chem. Theory Comput..

[23] Pickard C. J., Needs R. J. (2011). Ab initio random structure searching. J. Phys.: Condens. Matter.

[24] Buckingham R. A. (1938). The classical
equation of state of gaseous helium, neon, and argon. Proc. R. Soc. London A.

[25] Kohn W., Sham L. J. (1965). Self-Consistent Equations Including Exchange and Correlation
Effects. Phys. Rev. A.

[26] Li Z., Scheraga H. A. (1987). Monte Carlo-minimization
approach to the multiple-minima
problem in protein folding. Proc. Natl. Acad.
Sci. U. S. A..

[27] Wales D. J., Doye J. P. K. (1997). Global Optimization by Basin-Hopping and the Lowest
Energy Structures of Lennard-Jones Clusters Containing up to 110 Atoms. J. Phys. Chem. A.

[28] Collins C., Darling G. R., Rosseinsky M. J. (2018). The Flexible Unit Structure Engine
(FUSE) for probe structure-based composition prediction. Faraday Discuss..

[29] Price S. L., Leslie M., Welch G. W. A., Habgood M., Price L. S., Karamertzanis P. G., Day G. M. (2010). Modelling organic crystal structures
using distributed multipole and polarizability-based model intermolecular
potentials. Phys. Chem. Chem. Phys..

[30] Pyzer-Knapp E. O., Thompson H. P. G., Day G. M. (2016). An optimized
intermolecular force
field for hydrogen-bonded organic molecular crystals using atomic
multipole electrostatics. Acta Crystallogr.
B.

[31] Cui P., McMahon D. P., Spackman P. R., Alston B. M., Little M. A., Day G. M., Cooper A. I. (2019). Mining
predicted crystal structure
landscapes with high throughput crystallisation: old molecules, new
insights. Chemical Science.

[32] Spek A. L. (2009). Structure
validation in chemical crystallography. Acta
Crystallogr. B.

[33] Chisholm J. A., Motherwell S. (2005). COMPACK: a program for identifying
crystal structure
similarity using distances. J. Appl. Crystallogr..

[34] Sykes R. A., Johnson N. T., Kingsbury C. J., Harter J., Maloney A. G. P., Sugden I. J., Ward S. C., Bruno I. J., Adcock S. A., Wood P. A., McCabe P., Moldovan A. A., Atkinson F., Giangreco I., Cole J. C. (2024). What has scripting ever done for
us? The CSD Python application programming interface (API). J. Appl. Crystallogr..

[35] Sosoe J. O. E., Maris T., Wuest J. D. (2023). Strongly Hydrogen-Bonded
Networks
Formed by Sulfate and Bisulfate Salts of Benzenetetramines. Cryst. Growth Des..

[36] Martin J., Ceriotti M., Day G. M. (2025). An Adapted Similarity Kernel and
Generalized Convex Hull for Molecular Crystal Structure Prediction. Cryst. Growth Des..

[37] Chan K.-L., Yuen K.-H., Takayanagi H., Janadasa S., Peh K.-K. (1997). Polymorphism
of artemisinin from Artemisia annua. Phytochemistry.

[38] Brock C. P. (2022). Pervasive
approximate periodic symmetry in organic P1 structures. Acta Crystallogr. B.

