# Supplementary material for: Accelerating Prediction of Complex Molecular Crystals by Sensible Selection of Asymmetric Units
Source: J Chem Theory Comput. 2026 Jun 23;22(13):6986–99. doi: 10.1021/acs.jctc.6c00623 (PMC13374018; doi:10.1021/acs.jctc.6c00623)
Supplement: Supplementary file 1 [file ct6c00623_si_001.pdf]

Supplementary Information for: Accelerating Prediction of Complex Molecular Crystals by Sensible Selection of Asymmetric Units

Jordan A. Dorrell and Graeme M. Day*

School of Chemistry and Chemical Engineering, University of Southampton, Southampton, SO17 1BJ, UK

E-mail: g.m.day@soton.ac.uk

Space Group Symmetry Operators

UC2AU requires sampling of unit cells in space groups with a number of symmetry operators which is equal to the target Z'. We report in tables S1, S2, and S3 the number of symmetry operators for the space groups most commonly observed in the CSD.[1]

Table S1: Number of symmetry operators for top 10 most common space groups.

No. symmetry operators	No. space groups	Space groups
1	0	-
2	2	2, 4
3	0	-
4	6	14, 19, 33, 9, 29, 5
6	0	-
8	2	61, 16
16	0	-
18	0	-

Table S2: Number of symmetry operators for top 26 most common space groups.

No. symmetry operators	No. space groups	Space groups
1	1	1
2	3	2, 4, 7
3	1	145
4	9	14, 19, 33, 9, 29, 5, 18, 76, 13
6	1	169
8	7	61, 15, 60, 96, 56, 20, 86
16	2	43, 88,
18	1	148

Table S3: Number of symmetry operators for top 26 most common space groups. (any setting)

No. symmetry operators	No. space groups	Space groups
1	1	1
2	3	2, 4, 7
3	1	145
4	9	14, 19, 33, 9, 29, 5, 18, 76, 13
6	1	169, 148
8	7	61, 15, 60, 96, 56, 20, 86
16	2	43, 88,
18	1	148

Benchmarking

AUT and UC2AU have been benchmarked against a diverse range of systems. These are discussed in the main body of this work, but we report here the absolute values for the number of crystals (table S4), errors (table S5), and experimentally-observed crystals (table S6) yielded by each test. The core hours committed to each benchmark is constant across the structure generation algorithms (QRSS, AUT, or UC2AU) but varies between systems, with more complex systems receiving a larger allocation of compute time.

Table S4: Number of crystals acquisition of geometry optimised crystals by AUT and UC2AU with respect to QRSS. Rate for UC2AU represented with a '-' where the method is not applicable (Z'=1).

System	Z'	G	Space Group	QRSS	AUT	UC2AU
p-cresol	2	2	14	133290	139176	124194
p-cresol	3	3	15	38178	45355	46175
artemisinin	4	4	1	53943	73618	69958
pyridine	4	4	33	72557	81321	78757
BTA (bi)sulfate	1	3	9	104143	136326	-
BTA (bi)sulfate	1	4	7 (13)	86345	105388	-
BTA sulfate	1	5	4 (12)	70204	86614	-
TT.Br	1	4	15	56245	63223	-

Table S5: Relative frequency (errors per crystal) of errors during crystal generation (Gen) and geometry optimisation (Opt) by AUT and UC2AU with respect to QRSS. Errors during generation are largely attributed to crystals exceeding the volume tolerance. Errors during geometry optimisation are largely attributed to a failure to converge and Buckingham catastrophes. Rate for UC2AU represented with a '-' where the method is not applicable (Z'=1).

				QRSS		AUT		UC2AU	
System	Z'	G	Space Group	Gen	Opt	Gen	Opt	Gen	Opt
p-cresol	2	2	14	189922	22721	79642	18616	57993	16499
p-cresol	3	3	15	292579	16396	87613	10550	83751	10677
artemisinin	4	4	1	342113	12788	21505	4674	17525	4689
pyridine	4	4	33	313826	14186	32249	7402	23597	6564
BTA (bi)sulfate	1	3	9	218673	10802	48910	2250	-	-
BTA (bi)sulfate	1	4	7 (13)	320973	7442	37021	3133	-	-
BTA sulfate	1	5	4 (12)	446224	12717	24658	9922	-	-
TT.Br	1	4	15	350208	6099	116900	4951	-	-

Table S6: Absolute number of instances of discovery of experimentally-observed (or global minimum if none is available) crystals by QRSS, AUT and UC2AU. Rate for UC2AU represented with a '-' where the method is not applicable (Z'=1).

System	Z'	G	Space Group	QRSS	AUT	UC2AU
p-cresol	2	2	14	97	93	112
p-cresol	3	3	15	1	2	0
artemisinin	4	4	1	8	25	35
pyridine	4	4	33	7	27	35
BTA (bi)sulfate	1	3	9	7	127	-
BTA (bi)sulfate	1	4	7 (13)	14	108	-
BTA sulfate	1	5	4 (12)	10	11	-
TT.Br	1	4	15	1	7	-

References

(1) Groom, C. R.; Bruno, I. J.; Lightfoot, M. P.; Ward, S. C. The Cambridge Structural Database. *Acta Cryst* **2016**, *B72*, 171–179.
